# A Pilot Randomized, Placebo Controlled, Double Blind Phase I Trial of the Novel SIRT1 Activator SRT2104 in Elderly Volunteers

**DOI:** 10.1371/journal.pone.0051395

**Published:** 2012-12-20

**Authors:** Vincenzo Libri, Andrew P. Brown, Giulio Gambarota, Jonathan Haddad, Gregory S. Shields, Helen Dawes, David J. Pinato, Ethan Hoffman, Peter J. Elliot, George P. Vlasuk, Eric Jacobson, Martin R. Wilkins, Paul M. Matthews

**Affiliations:** 1 The National Institute for Health Research (NIHR)–Wellcome Trust Imperial College Clinical Research Facility, Imperial Centre for Translational and Experimental Medicine, Imperial College London, United Kingdom; 2 Imanova Centre for Imaging Sciences, London, United Kingdom; 3 GSK Clinical Imaging Centre, GlaxoSmithKline, Imperial College, London, United Kingdom; 4 Sirtris Pharmaceuticals, A GSK Company, Cambridge, Massachusetts, United States of America; 5 Movement Sciences Group, Oxford Brookes University, Oxford, United Kingdom; 6 Division of Brain Sciences, Department of Medicine, Imperial College, London, United Kingdom; Institut Pluridisciplinaire Hubert Curien, France

## Abstract

**Background:**

SRT2104 has been developed as a selective small molecule activator of SIRT1, a NAD^+^-dependent deacetylase involved in the regulation of energy homeostasis and the modulation of various metabolic pathways, including glucose metabolism, oxidative stress and lipid metabolism. SIRT1 has been suggested as putative therapeutic target in multiple age-related diseases including type 2 diabetes and dyslipidemias. We report the first clinical trial of SRT2104 in elderly volunteers.

**Methods:**

Oral doses of 0.5 or 2.0 g SRT2104 or matching placebo were administered once daily for 28 days. Pharmacokinetic samples were collected through 24 hours post-dose on days 1 and 28. Multiple pharmacodynamic endpoints were explored with oral glucose tolerance tests (OGTT), serum lipid profiles, magnetic resonance imaging (MRI) for assessment of whole body visceral and subcutaneous fat, maximal aerobic capacity test and muscle 31P magnetic resonance spectroscopy (MRS) for estimation of mitochondrial oxidative capacity.

**Results:**

SRT2104 was generally safe and well tolerated. Pharmacokinetic exposure increased less than dose-proportionally. Mean Tmax was 2–4 hours with elimination half-life of 15–20 hours. Serum cholesterol, LDL levels and triglycerides decreased with treatment. No significant changes in OGTT responses were observed. 31P MRS showed trends for more rapid calculated adenosine diphosphate (ADP) and phosphocreatine (PCr) recoveries after exercise, consistent with increased mitochondrial oxidative phosphorylation.

**Conclusions:**

SRT2104 can be safely administered in elderly individuals and has biological effects in humans that are consistent with SIRT1 activation. The results of this study support further development of SRT2104 and may be useful in dose selection for future clinical trials in patients.

**Trial Registration:**

ClinicalTrials.gov NCT00964340

## Introduction

SIRT1 is one of a family of seven nicotinamide adenine dinucleotide (NAD^+^)-dependent, protein deacetylase enzymes (called sirtuins) that contribute to the regulation of body energy homeostasis as well as many other responses to cellular stress. SIRT1 is broadly expressed in virtually every tissue including adipose tissue, liver, pancreas and skeletal muscle where it acts as the mediator of multiple cellular signaling pathways through the deacetylation of target proteins [1], [2], [3], [4]. Increased SIRT1 expression has been suggested as a target for therapeutic activation in multiple age- related diseases via the modulation of various metabolic pathways, including glucose metabolism [5], fatty acid oxidation [6], regulation of oxidative stress [7], lipid metabolism and fat mobilization in white adipocytes [8], [9], as well as improved insulin secretion [1], pancreatic β-cell preservation [9], [10] and increased insulin sensitivity [3], [9], [11].

The rationale for the pharmacological activation of SIRT1 by small molecules is based on beneficial pharmacology observed in animal studies where SIRT1 is genetically overexpressed or up-regulated due to calorie restriction [1], [2]. The polyphenolic compound resveratrol was the first compound shown to increase or activate SIRT1. However, due to its poor bioavailability, low potency and lack of specificity for SIRT1, resveratrol is not practical as a therapeutic [3], [4], [12], [13]. SRT2104 is the first generation of non-resveratrol compounds with improved drug-like properties that are more specific and potent synthetic direct activators of SIRT1 compared to resveratrol [3], [4], [5], [14], [15], [16].

*In-vitro* and animal studies have been performed to evaluate the pharmacologic and toxicologic properties of SRT2104 [17], [18]. The pre-clinical safety of SRT2104 has been investigated in the bacterial reverse mutation assay (AMES test), mouse lymphoma and mouse micronucleus genetic toxicology models, and in safety pharmacology studies in rats and dogs. SRT2104 was not genotoxic and was not associated with adverse central nervous system, cardiovascular system, or pulmonary effects in these preclinical safety and pharmacology studies. In vitro studies in human liver microsomes and cultured hepatocytes suggest that SRT2104 does not inhibit CYP1A, CYP2C9, CYP2C19, CYP2D6, and CYP3A4, or significantly induce cytochrome P450 isoforms CYP1A and CYP3A4.

SRT2104 was well tolerated and safe in young healthy volunteers for up to 7 days dosing of 0.03–3.0 g/day [19]. The elimination half-life (t_1/2_) was similar for doses up to 3.0 g/day, although increases in exposure were less than dose proportional at doses greater than 1.0 g/day.

No clinical data had been collected to date in elderly individuals who would be expected to be among the target populations for the treatment of many diseases of aging. Therefore, the primary objective of this study was to investigate the safety, tolerability and pharmacokinetic properties of SRT2104 when administered for 28 consecutive days in elderly male and female volunteers. In addition, we evaluated multiple exploratory pharmacodynamic endpoints to test whether the compound showed metabolic effects in humans that are consistent with those expected for SIRT1 activation.

## Methods

The protocol for this trial and supporting CONSORT checklist are available as supporting information; see Checklist S1 and Protocol S1.

### Study Participants, Design and Treatment

This was a phase I, single-center, double-blind, randomized, placebo-controlled, parallel-arm study to assess the safety, tolerability, pharmacokinetic and initial pharmacodynamic properties of SRT2104 (Sirtris Pharmaceuticals, Cambridge, MA, USA) administered for the first time to male and female elderly volunteers 60 to 80 years of age. Volunteers were enrolled between 01 Oct 2009 and 5 Feb 2010. Doses of 0.5 and 2.0 g or matching placebo were administered once daily for 28 consecutive days. The selection of the doses investigated in this study was based upon safety and pharmacokinetic data obtained from a previous clinical study involving SRT2104 [19]. Suitability of participants was defined on the basis of a medical history, physical examination, vital signs, electrocardiogram and laboratory measurements. Subjects were required to have normal fasting glucose levels (4.4 to 6.0 mmol/L) at screening and a body mass index of at least 18 kg/m^2^ and no greater than 30 kg/m^2^. Subjects were ineligible if they had a history of any chronic disease or any clinically significant illness within 3 months of study entry, including any history of renal or liver impairment and/or any endocrine, inflammatory, cardiovascular, gastro-intestinal, neurological, psychiatric, neoplastic or metabolic disease which in the opinion of the investigators could risk subject safety or interpretation of the results. Subjects were deemed ineligible if they participated in any clinical trial with an investigative medicinal product within the past three months prior to the first dose in the current study, had been exposed to more than three new chemical entities within 12 months of enrollment, or used other concomitant medications and herbal products in the previous 3 months that in the opinion of the investigator could interfere with the study procedures or compromise subject safety. Additional exclusions included: women of childbearing potential and non-sterile men, unless they agreed to use acceptable method of contraception; history of alcoholism or drug abuse (including a positive pre-study drug/alcohol test at screening); use of tobacco or nicotine products; history of complications when donating blood or known relative inaccessibility of veins for venipuncture; donation of blood within three months of enrolment; history of significant drug or other allergies. Participants were asked to maintain their usual level of physical activity for the duration of the study.

Twenty-four participants were planned to 3 parallel treatment arms (SRT2104 0.5 g/day, SRT2104 2.0 g/day, or placebo) and were randomized in a 1∶1∶1 ratio to provide evaluable data from 8 subjects in each of the 3 dose panels [see Methods S1 for subject random allocation sequence and un-blinding procedures]. There was no formal calculation of power for this study. A sample size of 8 subjects per group was chosen based on feasibility to allow preliminary characterisation of the safety, tolerability and pharmacokinetics of SRT2104 in the elderly population. In addition, a series of pharmacodynamic measures (see below) were included as secondary endpoints to preliminary assess the potential biological activity of the compound in humans.

Subjects participated in the study for approximately 79 days and underwent 2 screening visits, 2 inpatient visits, 7 outpatient visits and 8 telephone call visits (Figure S1). Subjects were screened during the 21 days prior to receiving their first dose of study drug (or placebo). They were admitted overnight as inpatients on the evenings prior to dosing on Days 1 and 28 for intense pharmacokinetic assessment. Subjects also returned to the clinical unit as outpatients on Days 7, 14 and 21 for weekly safety visits. Additional telephone safety assessments were made approximately on Days 3, 5, 10, 17, 20 and 24. The end of dosing follow-up visit was performed approximately 35 days following the first dose of SRT2104 or placebo. An additional follow up safety telephone call (defined as the last subject's last assessment) was made to each subject approximately 30 days following his or her final dose of SRT2104 and/or placebo.

During the treatment phase of the study (days 1 to 28 inclusive), test material (SRT2104 or placebo) was supplied as 250 mg capsules and was administered at approximately the same time every morning, approximately 15 minutes following consumption of a standardized meal (Ensure Plus®, a high energy, high protein liquid meal replacement product providing approximately 650 kcal with approximately 30% of calories derived from fat). Subjects were not permitted to consume additional calories for at least 1–2 hours after dosing, although water was permitted *ad libitum*. Subjects were also instructed to refrain from caffeine and alcohol for 24 hours prior to screening and during assessments visits. The test material was administered within the investigation clinical unit on the days when serial pharmacokinetic sampling was performed (days 1–2 and 28–29) and at home on the remaining days (days 3–27). A diary card was provided to volunteers to confirm daily consumption of the standardized meal, as well as time of dose and number of capsules taken. Missing doses were recorded.

At every visit (including at screening and follow-up), blood and urine samples were collected for clinical laboratory assessments (general blood biochemistry, full blood count, coagulation parameters and urinalysis). On the same days, physical examination, vital signs (resting pulse rate, respiration rate, temperature, and blood pressure readings), pre- and post-dose 12-lead ECG and a review of adverse events (AE) and any medications received were also performed. Safety evaluations were based on the incidence, severity and type of AEs and clinically significant changes from baseline in physical examination findings, vital signs or clinical laboratory results.

The study was approved by the Institutional Review Board of Imperial College Healthcare NHS Trust, London (UK), the UK Medicines and Healthcare products Regulatory Agency (MHRA; EudraCT number 2009-011918-21) and the Research Ethics Committee of Berkshire, Reading, UK (REC reference number 09/H0505/96). The study was registered on the public database ClinicalTrials.gov (reference number NCT00964340) and was conducted at The National Institute for Health Research (NIHR)-Wellcome Trust Imperial College Clinical Research Facility [formerly known as Sir John McMichael Clinical Research Centre (SJMC), Imperial College, London, UK] in compliance with the Declaration of Helsinki and the International Conference on Harmonisation (ICH) and Good Clinical Practice (GCP) guidelines. Written informed consent was obtained from each subject prior to the performance of any study-specific procedures. Subject's ability to consent was confirmed by medically qualified professionals of the study team.

### Bioanalysis and pharmacokinetic data analysis

Pharmacokinetic samples were collected through 24 hours post-dose on the first and last day of the dosing period (immediately prior to dosing and at 15, 30 minutes and 1, 2, 3, 4, 8, 12 and 24 hours post-dose). Blood samples for pharmacokinetic analysis were collected in pre-labelled lithium heparin heparin Vacutainers for pharmacokinetic analysis (∼4 mL per draw). Each sample was separated by centrifugation at 1500×g and 4°C for 10 minutes. Two equal aliquots of plasma were transferred to polypropylene vials labelled identically to the original blood sample and stored at approximately −20°C for subsequent analysis of plasma SRT2104 concentration. The bio-analysis was performed by Simbec Research Ltd, Merthyl Tydfil, South Wales, UK. Pharmacokinetic parameters for SRT2104 were derived from non-compartmental methods using WinNonLin v5.2 (Pharsight Corporation, Mountain View, CA, USA). SRT2104 concentrations were quantified in all subjects above a limit of quantification [LOQ] = 0.5 ng/mL. Actual blood sample collection times were used in the analysis. The maximum SRT2104 plasma concentration (C_max_) and the corresponding time of peak plasma concentration (T_max_) were taken directly from the individual plasma data. The mean area under the plasma concentration-time curve from time 0–24 hours (AUC_0–24_), the area under the plasma concentration-time curve from zero to time AUC(_0-τ_) (where τ is the time of the last measurable concentration) and the area under the plasma concentration-time curve from zero to infinity AUC(_0-∞_) were calculated by the linear trapezoidal rule. The terminal phase elimination rate constant (K_el_) was determined from the slope of the concentration-versus-time data plot during the log-linear terminal phase by regression analysis and the t_1/2_ was generated by dividing ln2 by the elimination rate constant K_el_. The accumulation ratio (R) was calculated as the ratio of day 28 to day 1 AUC_0–24_.

### Pharmacodynamic data analysis

#### Modified Oral Glucose Tolerance Test (mOGTT)

The oral glucose tolerance test (OGTT) allows identification and monitoring of individuals with impaired glucose tolerance and to evaluate the pharmacological effects of glucose lowering agents [20], [21]. A mOGTT was performed at baseline and on day 29 to assess the pharmacodynamic effects of SRT2104 (or placebo) on glucose, insulin and C-peptide levels. On both occasion, after an overnight fast, subjects were asked to drink a standard glucose beverage containing 75 g of glucose. Blood sample were drawn 10 min before and just prior to the subject consuming the beverage. Additional blood samples were drawn at 10, 20, 30, 60, 90, 120 and 180 minutes after the subject had consumed the glucose beverage. Plasma level of glucose, insulin and C-peptide were measured at each time point. The maximum glucose level (Gmax) was determined, and the area under the plasma glucose concentration–time curve (AUCgluc) was calculated using the trapezoidal rule. AUCgluc60 was defined as the area under the curve from 0 to 60 min after glucose ingestion, the period during which plasma glucose concentration increases. The effects of SRT2104 (or placebo) on glucose, insulin and C-peptide levels and relative AUCs were calculated in each subject as the differences between values at baseline (screening) and after 28 daily administrations of the test material.

#### ^31^P Magnetic Resonance Spectroscopy

Phosphorus-31 magnetic resonance spectroscopy (^31^P MRS) is a noninvasive tool for quantitatively monitoring the high-energy phosphate metabolism of skeletal muscle during exercise and recovery [22], [23], [24], [25]. Muscle concentrations of phosphocreatine (PCr), adenosine triphosphate (ATP), inorganic phosphate (Pi), and pH measured by ^31^P MRS are comparable to those measured by invasive biochemical analysis after biopsy [26]. ^31^P MRS experiments were performed on a clinical Siemens 3T Tim Trio (Siemens Healthcare, Erlangen, Germany). Subjects lay supine in the scanner. A custom-built ^31^P surface coil of 8 cm diameter was placed over the largest part of the gastrocnemius muscle. The measurement protocol consisted of a resting period of 2 min 8 s, an exercise period of 3 min 12 s, followed by a resting/recovery period of 5 min 20 s. During the exercise period, subjects performed repetitive plantar flexion of the foot against a custom-built, weighted pedal device at the rate of 30 repetitions per minute. Weighting of the pedal device during the exercise period was adjusted to be equivalent to 10–15% of lean body mass, as calculated with a height, weight and gender algorithm, using the formula of Hume [27]. ^31^P MR spectra were acquired with a “pulse-acquire” sequence. Each spectrum was generated by averaging 8 free induction decays (FID) acquired with a 2 s repetition time, which resulted in a 16 s time resolution per spectrum. Free induction decays were acquired with a dwell time of 0.5 ms and 1024 readout points. Spectra were analyzed using the AMARES routine of the jMRUI software package [28]. After manual phase-correction of the spectra, the PCr resonance peak was fitted to a Lorentzian lineshape to calculate tissue concentration. Intracellular pH was determined from the chemical shift of PCr and inorganic phosphate, and the value of ADP was calculated as in Vanderthommen et al. 2003 [29].

Analyses of ^31^P MRS measures of the T_1/2_ for ADP and PCr recovery (seconds) in the gastrocnemius muscle after exercise were assessed as changes from baseline to Day 27 using an ANCOVA model with treatment group as a factor and baseline as a covariate. No adjustment was made for multiple comparisons in consideration of the two estimates.

Recovery times of ADP and PCr were calculated by fitting a mono-exponential curve to the observed recovery data. The effects of SRT2104 (or placebo) on glucose, insulin and C-peptide levels and relative AUCs were calculated in each subject as the differences between values at baseline (screening) and after 28 daily administrations of the test material.

#### Magnetic Resonance Imaging

Magnetic Resonance Imaging (MRI) was also carried out on a clinical Siemens 3T Tim Trio (Siemens Healthcare, Erlangen, Germany). The torso was imaged using the 6-channel spine array and two 4-channel body array coils. The data were acquired using a 3D spoiled gradient echo sequence with partial Fourier acquisitions (VIBE) [30]. The imaging volume consisted of contiguous 3D slabs (voxel size of 1.4×1.4×5 mm3) acquired in a single 15 s end-expiration breath-hold (repetition time = 7 ms; echo times TEs = 2.45, 3.67 ms; flip angle = 10°; 450 mm field-of-view; GRAPPA factor 2). The most inferior slab was positioned at the pubic symphysis and the most superior slab surpassed the top of the lungs. Fat-only images in the abdomen were manually segmented into intra-abdominal adipose tissue (IAT) and abdominal subcutaneous adipose tissue (SAT). The abdominal area was defined by using anatomical landmarks (top of the femoral heads to the bottom of the right lung) [31]. All images were anonymized and blinded to time point, but not to subject in order to facilitate matching anatomical landmarks, and sent to a third party (Vardis Group Inc, London), who derived the adipose tissue volumes using Slice-O-Matic (Tomovision, Montreal, Canada) software. Analysis of compartmental fat distribution was performed for subcutaneous adipose tissue (SAT), visceral adipose tissue (VAT), and the ratio of SAT:VAT, using an ANOVA model with treatment group as a factor.

#### Maximal Aerobic Capacity Test

A stepwise incremental exercise test was performed on a cycle ergometer (Monarch 874E, Monark Exercise AB, Vansbro, Sweden) [32]. All participants were familiarized to the test procedure on a separate occasion prior to testing. Participants were seated in a standardized position. The test started with unloaded cycling, subjects were asked to maintain a cadence of 50 revolutions per minute (rpm), if the subject could not achieve 50 rpm they were encouraged to cycle as fast as the could (but not less than 40 rpm). Every two minutes the external load on the cycle ergometer was increased by 0.5 kg, this equated to a 25-Watt increase in workload at 50 rpm. Cardiac monitoring of heart rate, respiratory rate/minute ventilation and pulse oximeter for oxygen saturation were continuously recorded during the test.

Analysis of Maximal Aerobic Capacity parameters was performed using an ANOVA model with treatment group as a factor. Workload was progressed in 2-minute staged increments from an initial 3 minutes unloaded cycling, to exhaust subjects over a 6- to 10-min period. The test was terminated when the participant reached volitional exhaustion or their cadence dropped by 10 rpm. At the end of each increment, work-rate watts, rating of perceived exertion (Borg RPE scale), heart rate (Polar Vantage chest heart rate monitor, Polar Electro, Finland) and 3-lead ECG were recorded.

### Statistical methods

Analyses of demographics were performed on the Intent-to-treat (ITT) population including all subjects who were randomized. Analyses of exploratory pharmacodynamic measures were performed on the Per-Protocol (PP) population, including all randomized subjects who received study medication and had at least one post baseline efficacy assessment and no major protocol deviations.

Descriptive statistical analysis (N, mean, SD, CV%, median, minimum, and maximum, logarithmic transformation, analysis of variance (ANOVA) and 90% confidence intervals) was performed on pharmacokinetic parameters assessed at each sampling time point for each dose level. Natural log-transformed AUC_0–24_, AUC_0-∞_ and C_max_ data using a linear mixed effects model with dose level and gender as fixed effects and subject as a random effect were used to analyze differences between treatments. Statistical analysis of accumulation ratio was performed after a ln transformation of the data from each dose level. A mixed effect model was fitted with day and gender as fixed effects and subject as a random effect. Day 28 (last dosing day) was compared with day 1 (first dose) in order to estimate the accumulation ratio for each dose level. The ratio was calculated from the geometric least-square mean from day 28/day 1. Statistical significance was set at *P*≤0.05, 2-sided. Dunnett's test was used to adjust for multiple comparisons against placebo. The statistical analysis was performed using the SAS® for Windows software package (Version 9.1.3) (SAS Institute, Cary, North Carolina, USA).

## Results

This was a pilot Phase I study of non-therapeutic objectives to allow preliminary characterization of safety, tolerability and drug kinetics of SRT2104 in male and female elderly volunteer following single and repeat daily dosing for 28 days.

Sample size was not based upon formal calculation of power or regulatory guidelines but it was consistent with most first-in-man/Phase-I drug's pharmacokinetic trials designed to provide sufficient information about the drug's pharmacokinetics profile and allow the design of future Phase II trials. In addition, although the study was not designed to fully characterize the pharmacological effects of SRT2104, it evaluated the potential biological activity of the compound for the first time in humans, and that is an information that has not been previously reported. However, given the exploratory nature of our investigation, results should be interpreted with some caution and require further substantiation from larger confirmatory studies in adequately selected target populations.

### Demographics, Safety and Tolerability

**Figure 1:**
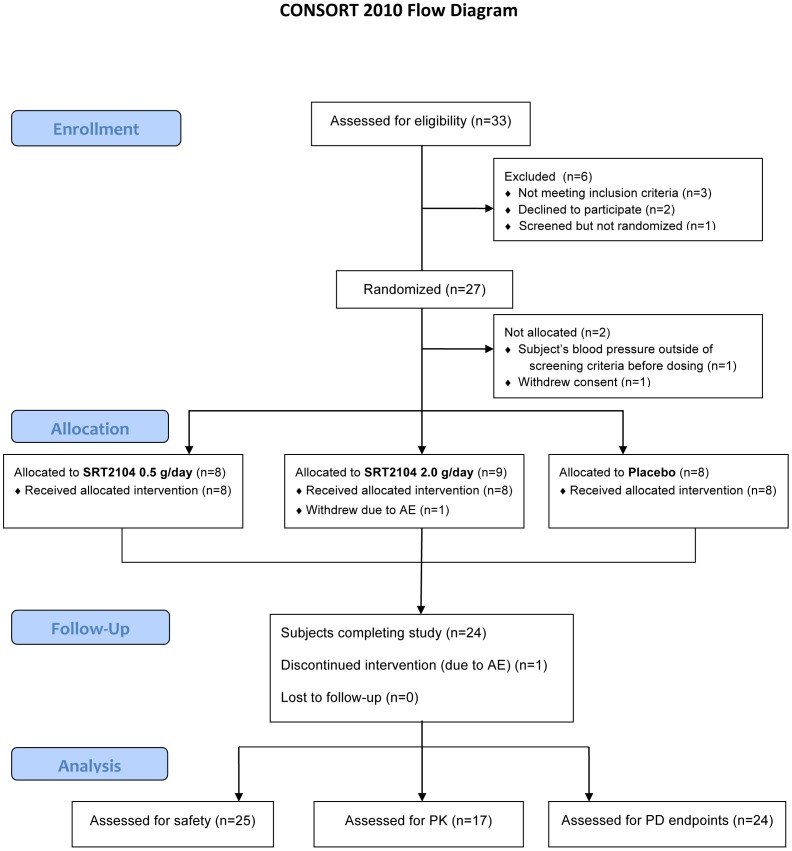
CONSORT 2010 Flow Diagram. The schema graphically outlines the design and conduct of the clinical study.

The CONSORT 2010 flow diagram of this study is shown in Figure 1. A total of 33 subjects were screened to take part in this study. Two subjects withdrew consent prior to being randomized, three were not considered eligible and one subject was screened as a ‘reserve’ and was not randomized into the study. Of the 27 subjects that were randomized into the study, two withdrew prior to receiving study medication and one subject was withdrawn after receiving study medication due to an adverse event (lower respiratory tract infection requiring treatment with amoxicillin).

The remaining 25 subjects were dosed with placebo (n = 8), SRT2104 0.5 g/day (n = 8) or SRT2104 2.0 g/day (n = 9). One subject who was assigned to the SRT2104 2.0 g/day regimen was withdrawn from the study on day 2 of the dosing period due to an acute chest infection, which was not believed to be related to SRT2104. For another subject in the SRT2104 0.5 g/day cohort, study drug was stopped on day 14 (due to increased serum creatinine) and restarted on day 21 (after normalization of creatinine concentration). All other subjects completed their planned dosing schedules. The ‘safety population’ included all subjects who were randomized and received at least one dose of study medication or placebo (n = 25). All cohorts were comparable at baseline with respect to race, sex, average age, weight and BMI (Table 1).

**Table 1:** Summary of subject demographics at screening.

Characteristic		Placebo	0.5 g/day	2.0 g/day	No Treatment	Total study group
Age (years)	N	8	8	9	2	27
	Mean (Min – Max)	67.0 (62–73)	67.6 (61–77)	66.2 (61–72	69.5 (69–70)	67.1 (61–77)
Sex	Female	4 (50%)	5 (63%)	6 (67%)	1 (50%)	16 (59%)
	Male	4 (50%)	3 (38%)	3 (33%)	1 (50%)	11 (41%)
Race	Asian	0	1 (13%)	0	0	1 (4%)
	Black	0	1 (13%)	0	0	1 (4%)
	White	8 (100%)	6 (75%)	9 (100%)	2 (100%)	25 (93%)
BMI	Kg/m^2^ (SD)	25.0 (3.24)	26.0 (1.31)	25.5 (2.97)	24.2 (1.27)	
Weight	Kg (SD)	73.6 (10.70)	68.8 (6.58)	67.3 (7.07)	63.1 (7.57)	

SD = Standard Deviation.

SRT2104 was generally well tolerated at both dose levels. No significant difference in the incidence and severity of adverse events (AE) were detected between treatment groups (including placebo) although a higher incidence of headache and nasopharyngitis was reported in the SRT2104 0.5 g/day and 2.0 g/day groups, respectively (Table 2). The latter (nasopharyngitis), however, was considered to have an unlike or no relationship to the study medication and it was more likely to be related to the time of the year (winter) during which the affected subjects participated in the study. The most frequently reported AE were predominantly gastro-intestinal in nature (including nausea, constipation, flatulence, and loose bowel movements) but their incidence was slightly higher in the placebo group relative to the active treatment groups. The majority of AE were mild-moderate in severity, short in duration and reversible without pharmacological intervention.

**Table 2:** Incidence of all treatment-emergent adverse events by subject treatment group.

Treatment	Placebo		SRT2104		SRT2104	
			0.5 g/day		2.0 g/day	
	(n = 8)		(n = 8)		(n = 9)	
Number of subjects experiencing any adverse event	8	(100%)	8	(100%)	9	(100%)
**Nervous system disorders**						
Headache	2	(25%)	5	(63%)	1	(11%)
Lethargy	1	(13%)	1	(13%)	0	
Dizziness	0		0		1	(11%)
Migraine	0		1	(13%)	0	
Syncope vasovagal	0		0		1	(11%)
**Gastrointestinal disorders**						
Diarrhoea	3	(38%)	1	(13%)	2	(22%)
Nausea	2	(25%)	1	(13%)	0	
Abdominal distension	1	(13%)	1	(13%)	0	
Abdominal pain lower	0		1	(13%)	0	
Constipation	0		0		1	(11%)
Vomiting	0		1	(13%)	0	
**Infections**						
Nasopharyngitis	0		1	(13%)	4	(44%)
Lower respiratory tract infection	0		0		1	(11%)
**General disorders**						
Application site haematoma	2	(25%)	1	(13%)	1	(11%)
Fatigue	1	(13%)	0		0	
Mass	1	(13%)	0		0	
Tenderness	1	(13%)	0		0	
**Injury and procedural complications**						
Thermal burn	1	(13%)	0		1	(11%)
Limb injury	0		0		1	(11%)
Procedural pain	0		1	(13%)	0	
Fall	1	(13%)	0		0	
**Musculoskeletal and connective tissue disorders**						
Arthralgia	0		1	(13%)	0	
Back pain	0		1	(13%)	0	
Sensation of heaviness	0		1	(13%)	0	
Joint stiffness	1	(13%)	0		0	
Muscle spasms	1	(13%)	0		0	
Muscular weakness	1	(13%)	0		0	
**Blood and urine**						
Blood creatinine increased	0		1	(13%)	0	
Urine output increased	0		1	(13%)	0	
**Psychiatric disorders**						
Insomnia	0		1	(13%)	0	
Sleep disorder	0		1	(13%)	0	
**Respiratory, thoracic and mediastinal disorders**						
Cough	0		0		1	(11%)
Dysphonia	0		0		1	(11%)
Epistaxis	1	(13%)	0		0	
**Skin and subcutaneous tissue disorders**						
Rash	1	(13%)	1	(13%)	0	
Erythema	0		1	(13%)	0	
**Cardiac disorders**						
Palpitations	0		1	(13%)	0	
**Eye disorders**						
Dry eye	1	(13%)	0		0	
Eye pain	1	(13%)	0		0	
**Metabolism and Nutrition Disorders**						
Decreased Appetite	1	(13%)	1	(13%)	0	

### Pharmacokinetics

**Figure 2:**
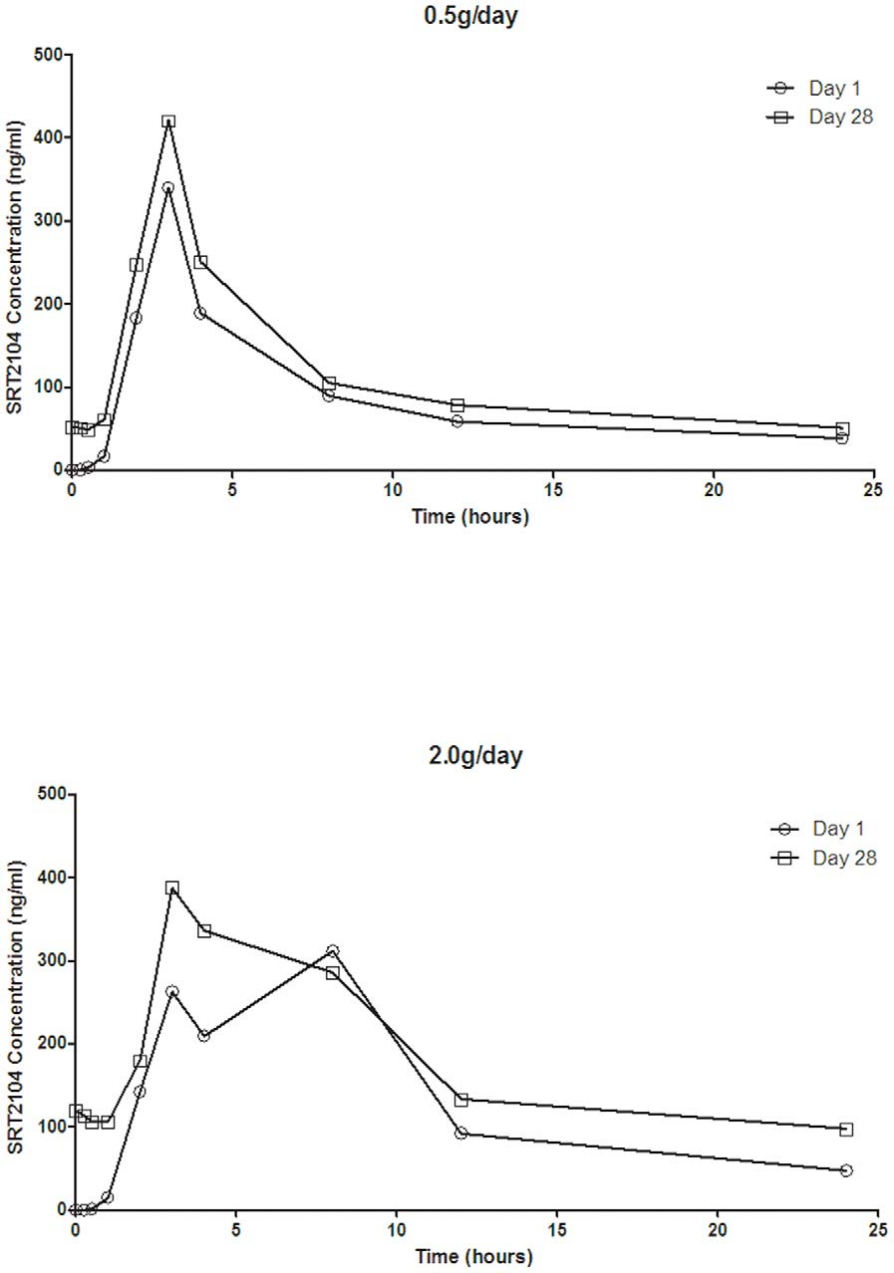
Mean plasma concentration versus time plots for SRT2104 on day 1 and 28, following multiple dose administration of 0.5 g/day (top panel) and 2.0 g/day (lower panel) to elderly male and female volunteers.

The ‘pharmacokinetic analysis population’ included all subjects who were randomized, and had received at least one dose of SRT2104 (n = 17). SRT2104 achieved mean peak plasma concentration (T_max_) at 2–4 hours in the 0.5 g/day group (Figure 2; Table 3). A longer apparent mean rise to peak concentration (T_max_ of 2–8 hours) was observed after the first dose in the 2.0 g/day group, but the day 28 plasma concentration kinetics for this group were similar to that seen at 0.5 g/day. Plasma concentrations subsequently declined in a mono-exponential manner with an apparent half-life of approximately 15 hours. No dose dependence was observed for either the T_max_ or elimination half-life (t_1/2_). The C_max_, AUC(_0-τ_) and AUC(_0–24_) values were less than dose proportional, only achieving exposures approximately two-fold higher or less in the 2.0 g/day group compared to the 0.5 g/day group.

**Table 3:** Summary of derived SRT2104 pharmacokinetic parameters by dose level.

Dose	Day		C_max_	t_max_ 1	AUC_0-t_	AUC_0-∞_ 2	t_1/2_	CL/F
			(ng/mL)	(hr)	(ng/ml.hr)	(ng/ml.hr)	(hr)	(L/hr)
0.5 g/day	1	N	8	8	8	8	8	8
		Mean	343.4	3.0	1872.9	1790.8	14.9	594.2
		SD	232.4	0.5	1089.0	1584.1	6.9	1072.9
	28	N	8	8	8	7	7	8
		Mean	373.4	3.0	2280.4	2687.8	17.5	566.9
		SD	286.5	1.122	1585.9	2351.9	5.2	867.6
2.0 g/day	1	N	9	9	9	6	6	6
		Mean	477.6	3.0	3249.2	2073.5	15.1	1624.4
		SD	523.0	2.3	3236.0	2207.7	5.9	1814.6
	28	N	8	8	8	6	6	8
		Mean	572.4	3.0	4797.4	7729.6	21.6	789.6
		SD	353.0	2.0	2579.9	3478.6	12.5	1002.0

1 Median is presented for t_max_.

2 Geometric mean is presented for AUC_0-∞_.

Abbreviations:

C_max_ = maximum plasma concentration.

t_max_ = time of peak plasma concentration.

AUC_0-t_ = area under the plasma concentration-time curve from zero to the time of the last measurable concentration.

AUC_0-∞_ = area under the plasma concentration-time curve from zero to infinity.

t_1/2_ = half life.

CL/F = clearance.

Repeat administration of SRT2104 of 2.0 g/day resulted in approximately two-fold increases in the mean AUC(_0–24_) relative to the first day of dosing and a higher mean t_1/2_ on day 28 (21.6±12.5 hr) relative to day 1 (15.1±5.9 hr). Between-subject variability for AUC(_0–24_) was high for both dose groups. The geometric mean CV for the 0.5 g/day group was 140% and for the 2.0 g/day group was 102%. As described above, one subject who was assigned to the SRT2104 0.5 g/day cohort had study drug washed out starting at day 14 (due to increased serum creatinine levels of 151 µmol/L) and then restarted on day 21 of the dosing period (upon creatinine normalization, i.e. 126 µmol/L). Subsequent repeat measurements of serum creatinine levels revealed a relapse of out of range values (154 µmol/L on day 28) and a return to normal range (116 µmol/L) at the end of study follow up visit. Comparable pharmacokinetics were observed in this subject relative to the other subjects in the SRT2104 0.5 g/day cohort at the end of the 28 dosing period. Summaries of statistical analysis of Pharmacokinetic Data are presented in Table 4 (day 28 *vs.* day 1) and Table 5 (dose proportionality). No apparent differences in exposure were observed between men and women.

**Table 4:** Summary of Statistical Analysis of SRT2104 Pharmacokinetic Data: Day 28 vs Day 1.

		Geometric Least Square Means		
SRT2104 Dose Level	Parameter	Day 1	Day 28	Day 28/Day 1 (90% C.I.)
0.5 g/day	C_max_ (ng/ml)	326.22	199.12	61.04 (14.65–254.30)
	AUC_(0-t)_ (ng.h/ml)	1950.07	1610.10	82.57 (34.83–195.70)
	AUC_(0-∞)_ * (ng.h/ml)	2503.70	1612.40	64.40 (29.19–142.09)
2.0 g/day	C_max_ (ng/ml)	301.28	389.50	129.28 (40.01–417.76)
	AUC_(0-t)_ (ng.h/ml)	1743.14	3577.50	205.23 (79.36–530.77)
	AUC_(0-∞)_ * (ng.h/ml)	1788.42	3669.38	205.17 (88.52–475.58)

* AUC values included in the analysis are AUC_(0-∞)_ on Day 1 and AUC_(0-τ)_ on Day 28.

Results obtained from a mixed model ANOVA on log-transformed data with fixed effects of study day and gender and a random effect of subject.

**Table 5:** Summary of Statistical Analysis of SRT2104 Pharmacokinetic Data: Dose Proportionality.

Study Day		SRT2104 Dose Level		Geometric Least Square Means
		Geometric Least Square Means		% Ratio (90% C.I.)
	Dose-Normalized Parameter	0.5 g/day	2.0 g/day	2.0 g/0.5 g
Day 1	C_max__D (ng/ml)	215.74	69.64	32.28 (10.41–100.15)
	AUC_(0-t)__D (ng.h/ml)	1297.86	516.59	39.80 (14.39–110.07)
	AUC_(0-∞)__D (ng.h/ml)	1754.57	504.43	28.75 (9.56–86.49)
Day 28	C_max__D (ng/ml)	206.06	96.83	46.99 (15.44–142.99)
	AUC_(0-t)__D (ng.h/ml)	1548.09	911.01	58.85 (25.18–137.51)
	AUC_(0-τ)__D (ng.h/ml)	1548.74	909.85	58.75 (25.10–137.52)
	AUC_(0-∞)__D (ng.h/ml)	2636.12	1903.42	72.21 (33.02–157.88)

Results obtained from an ANOVA on dose-normalized (to 0.5 g) log-transformed data with fixed effects of dose level and gender.

### Exploratory Pharmacodynamic Measures

#### Serum Lipid Profile

**Figure 3:**
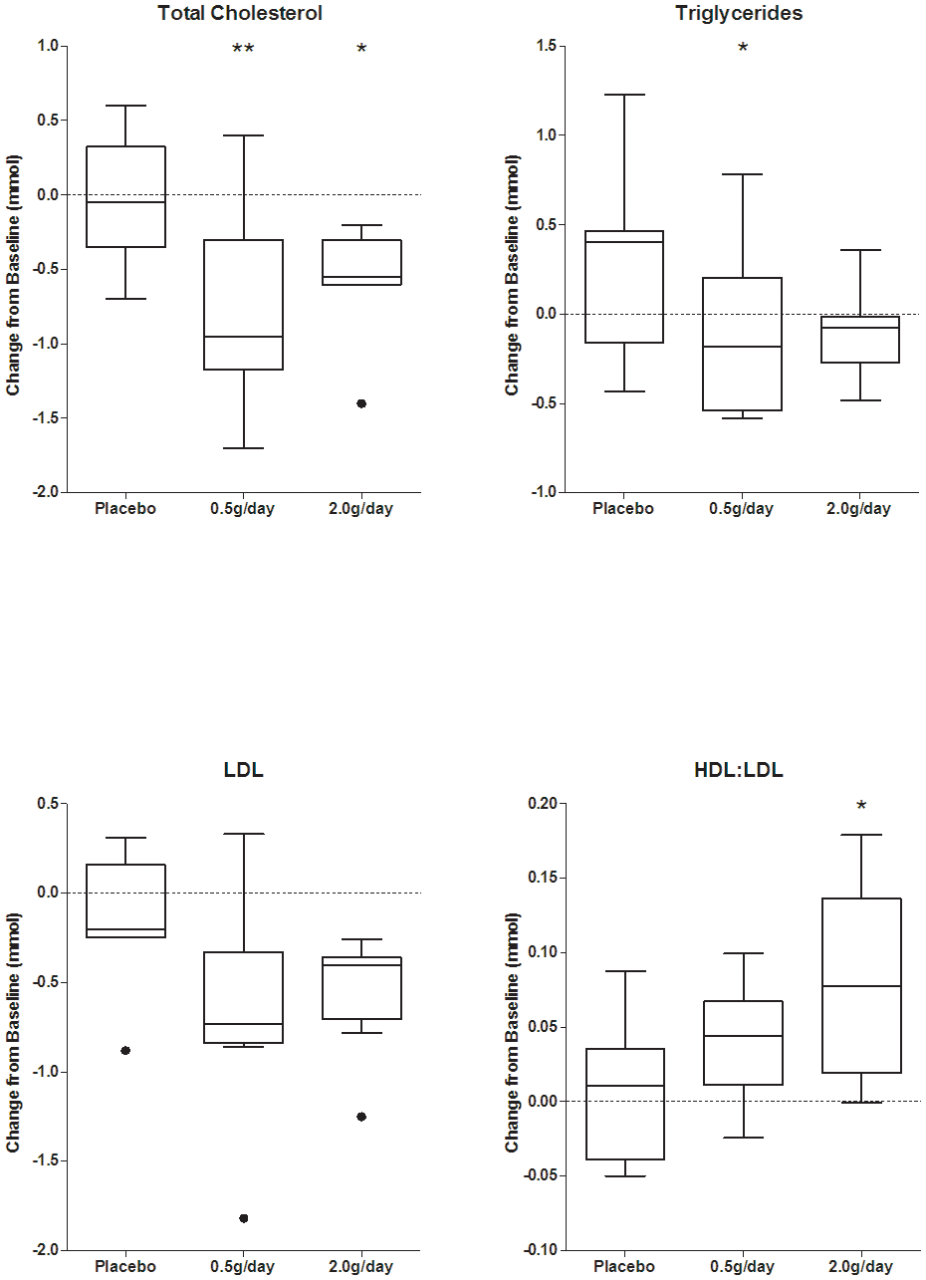
Lipid profile at baseline and after 28 days treatment with placebo and SRT2104 (0.5 g/day and 2.0 g/day). [Black dots indicate points that are two interquartile ranges outside the means; * P<0.05 and ** P<0.001 at day 28 relative to baseline.]

At the end of study treatment (day 28) there was a statistically significant decrease in serum cholesterol levels in both SRT2104 0.5 g/day and 2.0 g/day groups (p = 0.0071 and p = 0.0181, respectively), relative to baseline, as compared to placebo (Figure 3).

This was accompanied by decreases in low-density lipoprotein (LDL) cholesterol (but not changes in high-density lipoprotein, HDL) and a dose-dependent increase in the mean HDL:LDL ratios that was statistically significant for the SRT2104 2.0 g/day group (p = 0.0141), as compared to placebo. The decrease in total cholesterol and LDL levels as well as the increase in HDL:LDL ratio reverted to baseline values after 7 days of drug washout (Figures S2 and S3). Individual serum cholesterol levels at baseline and after 28-day treatment with SRT2104 0.5 g/day and 2.0 g/day are presented in Figure S4.

A decrease in mean serum triglyceride concentration also was observed with active treatment at day 28 relative to baseline, as compared to placebo, although this was not dose-dependent and was reflected only as a trend for the SRT2104 2.0 g/day dose group (Figure 3).

#### Oral glucose tolerance tests (OGTTs)

**Figure 4:**
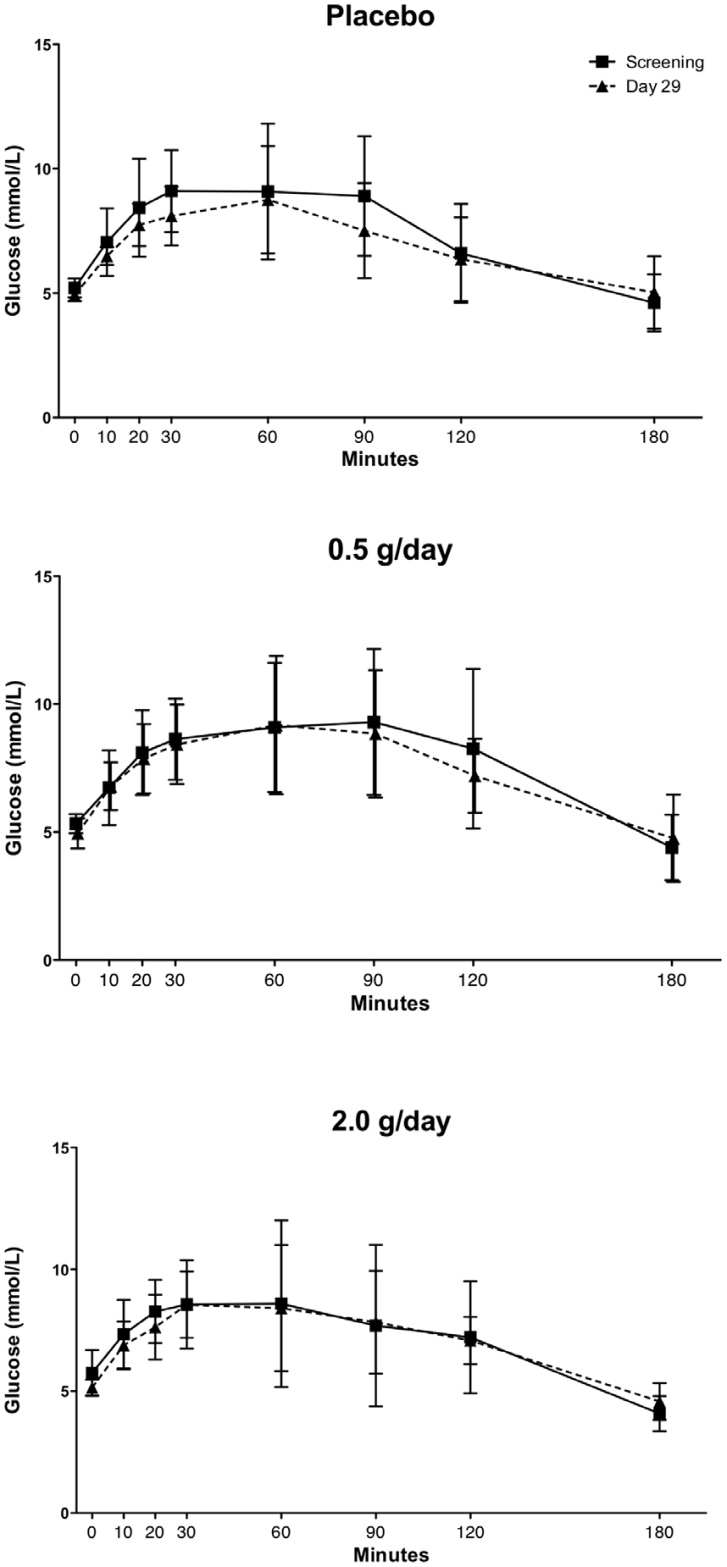
Oral glucose tolerance tests (OGTTs) at baseline and after 28 days treatment placebo (upper) SRT2104 0.5 g/day (middle) or SRT2104 2.0 g/day (lower panel).

**Figure 5:**
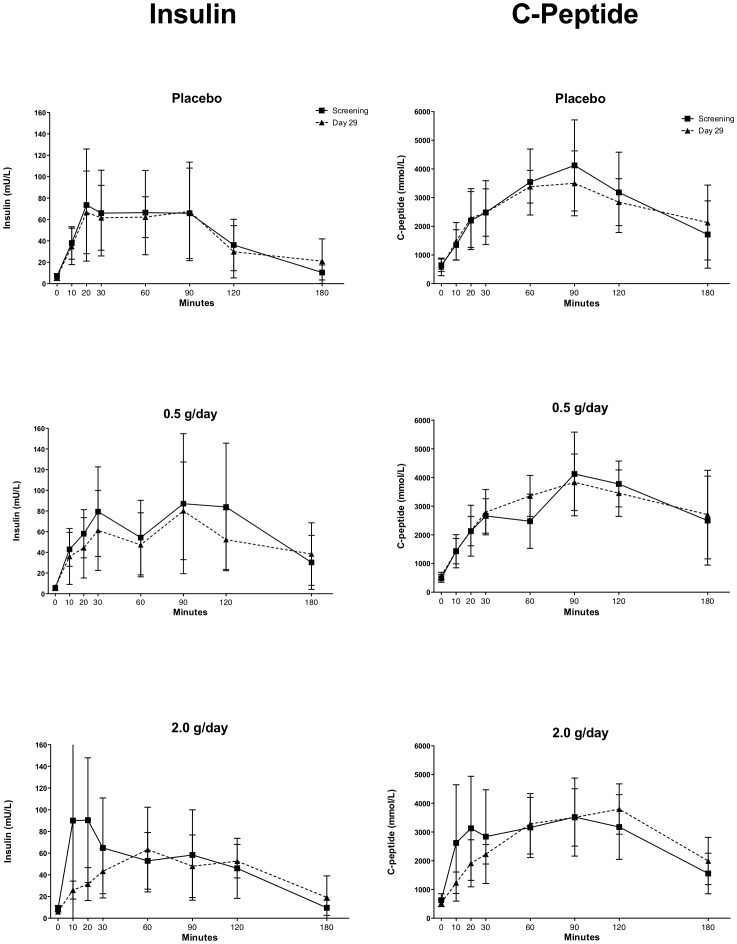
Insulin (left) and C-Peptide (right) concentration–time curves at baseline (screening) and after 28 days treatment with placebo (upper) SRT2104 0.5 g/day (middle) or SRT2104 2.0 g/day (lower panel).

All participants were enrolled into the study and randomized to treatment arms based on their normal glucose levels at screening (<6.0 mmol/L). They also underwent OGTTs prior to starting treatment and again on day 29 to assess the pharmacodynamic effects of SRT2104 on glucose, insulin and C-peptide levels.. Maximum serum glucose concentrations (Gmax) and area under the curve of glucose concentration–time (AUCGlu) were similar before and after either placebo or SRT2104 treatment (Figure 4). Likewise, no statistically significant changes in insulin and C-peptide levels and relative AUCs were observed in any of the treatment groups, although trends to reduced rates in the rise of both insulin and C-peptide and a lower peak insulin concentration were observed for the SRT2104 2.0 g/day group at day 29 relative to baseline (Figure 5).

#### 31P MRS, MRI and Maximal Aerobic Capacity Test

**Figure 6:**
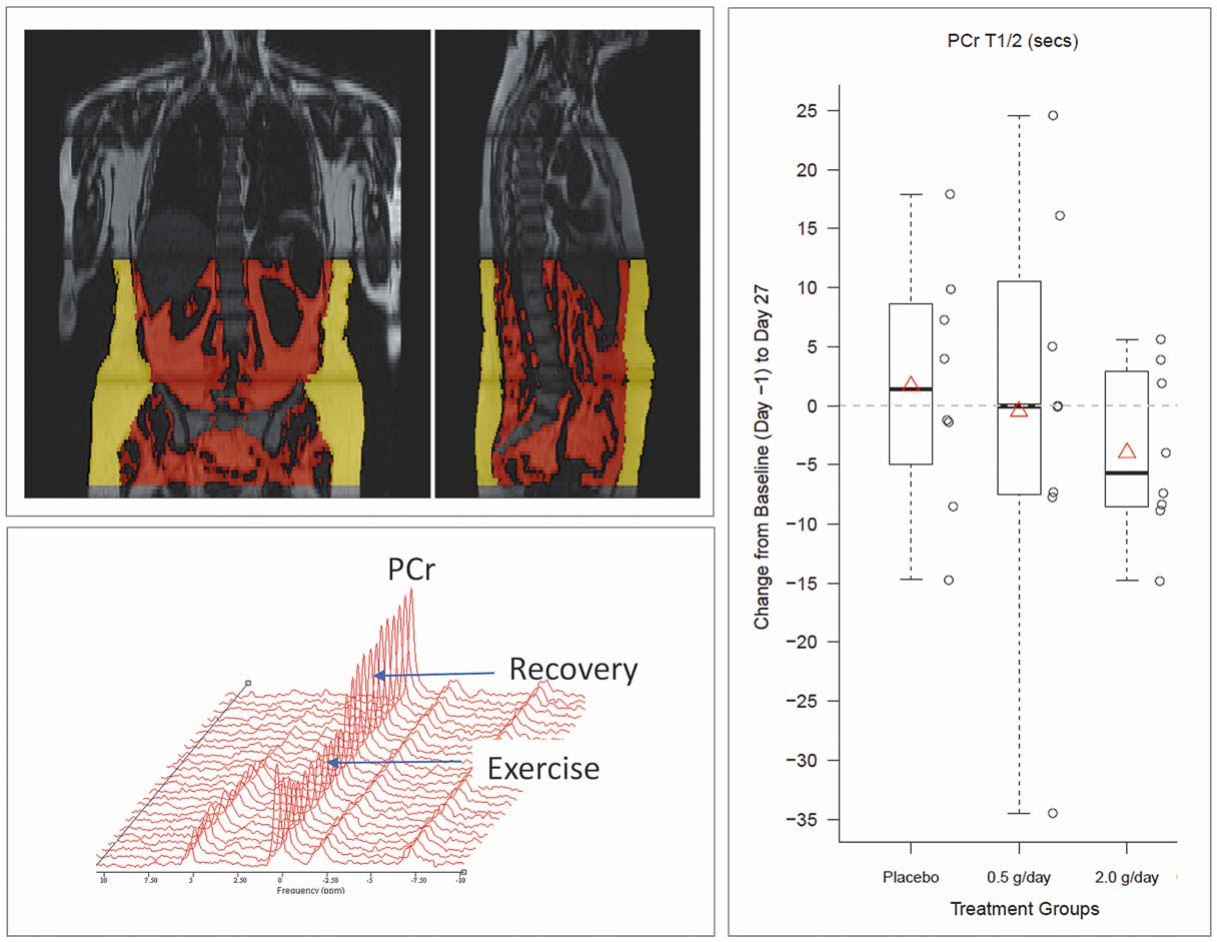
Representative example of magnetic resonance images and ^31^P Magnetic resonance spectroscopy results. Top left panel: intra-abdominal (red) and subcutaneous (yellow) adipose tissue maps overlaid on coronal and sagittal body MRI images from a single subject. Bottom left panel: dynamic series of ^31^P MRS spectra acquired serially during exercise and recovery from a single subject as described in Methods. Right panel: box plot of PCr recovery time change from baseline (day −1) to day 27 for the placebo, SRT2104 0.5 g/day and 2.0 g/day groups.

SRT2104 0.5 g/day and 2.0 g/day showed a trend for a decrease in half-time for recovery of the calculated adenosine diphosphate (ADP) concentration (ADP T_1/2_) values after exercise, on day 27 relative to baseline (Table S1). This effect was greatest in the SRT2104 2.0 g/day group (mean decrease of 15%). As there were no changes in resting pH or rates of pH recovery after exercise with treatment, a *post hoc* exploratory analysis was performed using the more direct measure of muscle mitochondrial oxidative metabolic capacity provided by the half-time for recovery of phosphocreatine (PCr) concentration (PCr T_1/2_) relative to baseline values after exercise. A trend for a decrease (mean decrease 14%) in PCr T_1/2_ after treatment was found for the SRT2104 2.0 g/day dose group relative to placebo (p = 0.083) (Figure 6 and Table S1).

Fat was distinguished on whole body MRI images and subcutaneous (SAT) and visceral (VAT) adipose tissue volumes were measured separately. No consistent changes from baseline were observed in either adipose tissue measures and in the VAT:SAT ratio with active treatment relative to placebo. Likewise, no consistent changes in exercise endurance were found with treatment. A small (4%) but statistically significant decrease in exercise capacity (as measured by time to cessation for the staged bicycle assessment) at day 27 was observed in the SRT2104 0.5 g/day group (p = 0.004), although there was no evidence for any changes in the 2.0 g/day group (Table S2).

## Discussion

We are reporting results of the first clinical study in elderly volunteers with SRT2104, a compound belonging to a series of novel SIRT1 activators being developed for the treatment of type II diabetes and other-age related diseases [14], [15]. There were no serious adverse events and the drug was generally well tolerated during 28 days continuous administration, with no dose-limiting toxicities being detected. However, given the exploratory nature of our study, larger, prospectively conducted studies are required to conclude on the long-term safety of SRT2104 in the general population.

Our data suggest a less than dose proportional increase in exposure for SRT2104 between 0.5–2.0 g/day. In a previous oral dose study in young healthy volunteers, AUC(_0-τ_) and C_max_ exhibited an approximate dose proportionality over the dose range 0.03 g/day to 1.0 g/day and a less than dose proportional increase between 1.0–3.0 g/day when administered in the fasted state [19]. Substantial inter-individual variability in systemic exposure was observed at both doses tested in our study, although this also was comparable to the pharmacokinetics observed previously in healthy young male subjects in the fasted state.

In a previous Phase 1 study in healthy young male volunteers [19], a substantial food effect was observed for this compound. When SRT2104 was administered with a standardized solid meal, an increase in overall exposure of SRT2104 was observed for both the 0.5 and 2.0 g doses, compared with administration in the fasted state. There was also a substantial reduction in inter-subject variability in exposure in the fed state. It was anticipated that the standardized meal of Ensure Plus® taken prior to dosing would simulate the effect of a solid meal because it contained a similar caloric content. However, the variability and exposure observed in the current study was similar to fasted-state exposure, rather than fed-state. A proportion of this pharmacokinetic variation may reflect individual differences in absorption metabolism, or a combination of the two; one subject in the 0.5 g/day group exhibited t_1/2_ values of greater than 24 hours after both first and repeated administrations (29.7 and 28.3 hours, respectively), although the C_max_, AUC(_0-τ_) and AUC(_0–24_) were considerably lower than for other subjects. However, other data (unpublished) suggests that exposure is inversely related to gastric emptying rate. A liquid meal of Ensure Plus®, while calorically similar to a standardized solid meal, may have accelerated gastric emptying, which may explain why a food-related increase in drug absorption was not observed in this study. Changes in individual pharmacokinetics may be just a consequence of differences in the ontogeny of drug elimination pathways [33].

Although this study was primarily designed to evaluate safety and pharmacokinetic outcomes, potential pharmacodynamic measures were also explored as secondary endpoints to preliminary assess the biological activity of the compound in humans. Effects of SRT2104 on plasma lipid profile were observed, including a decrease in serum cholesterol and triglycerides, as well as an increase in HDL:LDL ratio which appeared to be a consequence of decreases in pro-atherogenic LDL cholesterol. Notably, these effects increased through the dosing period and reversed to baseline (pre-treatment) values within one week of drug washout. Although subjects were instructed to maintain their standard level of physical activity and diet for the entire duration of the study, with the exception of consuming a standardized breakfast (Ensure Plus®) every morning approximately 15 minutes prior to dosing, we cannot exclude that a change in life style over a relatively short period of time may have acted as a potential confounder affecting subjects' cholesterolaemia. However, given the fact that changes in lipid profile were only observed in subjects receiving SRT2104 but not placebo seems to suggest a genuine drug-dependent effect consistent with target modulation. Both resveratrol and SRT2104 administration lower cholesterol, LDL and triglycerides in preclinical models of dyslipidemia, diabetes and obesity (e.g., DIO and Lep^ob/ob^ obese mice) [4], [17], [18], [34], [35]. These results in humans may be partially explained by a positive regulatory effect of SRT2104 on liver X receptor (LXR) a nuclear receptor involved in the regulation of cholesterol and lipid homeostasis [36].

The effects of SRT2104 on cholesterol levels seem also comparable to the antihyperlipidemic effects of niacin (nicotinic acid), at an intake of 1 g/day or higher [37], which is considered to be mediated by a change in intrahepatic LDL-triglyceride secretion and metabolism [38]. However, intakes of niacin at quantities of one gram or more carry significant risk of side effects (e.g., headache, nausea, vomiting, skin-flushing and liver function toxicity), which may require close monitoring, decreased dosage or discontinuation in favor of other agents. Further studies will be required to explore possible lipid lowering effects of SIRT1 activators in patients with type II diabetes and dyslipidemia, in whom the major targets for therapeutic modulation by SIRT1 may need to be different [39].

We did not observe significant changes in OGTT in this population of elderly individual treated with SRT2104, despite a trend toward a slower increase in insulin and C-peptide in the SRT2104 2.0 g/day treatment group. While the latter effect appears to be consistent with enhanced insulin sensitivity, a known consequence of SIRT1 activation, it remains to be proven in larger, well-selected cohorts of patients given that our observations were based on a very small sample size and short duration of treatment. In addition, the classification of subjects based on only a single OGTT is insecure [40], [41].

We further explored potential mechanisms mediating the possible increase in insulin sensitivity using ^31^P-MRS to test for a treatment-related increase in muscle mitochondrial oxidative metabolic capacity. Rates of high-energy phosphate recovery after exercise (decreasing the elevated calculated [ADP] and increasing the partially depleted [PCr] to resting muscle baseline values) provide an index of muscle mitochondrial oxidative metabolism [6] that correlates well with biochemical measures of maximum mitochondrial oxidative capacity [9]. Exercise-induced increases in muscle oxidative phosphorylative capacity are associated with increased SIRT1 expression [42], although effects of SIRT1 on mitochondria function are not limited to stimulation of biogenesis [43]. The calculated ADP recovery rate post-exercise (ADP T_1/2_) showed a trend for a small increase in the SRT2104 treatment groups. A *post hoc* analysis of PCr T_1/2_ (which potentially allows higher precision for estimation of the net oxidative phosphorylation rate, as it does not have a dependence on intracellular pH measurements from the chemical shift of muscle inorganic phosphate [6]) was consistent with this in showing a trend for dose-dependent increases.

However, we did not see improvements in measures of maximal aerobic capacity and exercise oxygen consumption (VO2max), which is also known to correlate well with PCr recovery kinetics [44]. This may be a consequence of a lack of study power or relative deconditioning and lack of a major dependence of aerobic exercise on metabolic adaptations in skeletal muscle training, which in this population of elderly subjects may have resulted in reduced mitochondrial enzyme activities, lipid oxidation and inability to decrease lactic acid accumulation [45], [46]. Interestingly, it has recently been reported that the O2 diffusing capacity is well preserved in the elderly whereas the age related decline in oxidative capacity is most likely a consequence of limited mitochondrial content and/or mitochondrial dysfunction rather than O2 availability [47].

Similarly, no significant difference in the MRI quantification of adipose tissue was noted in our study, despite possible beneficial effects on lipids. Weight loss in obesity and type diabetes is a likely longer-term effect of diet and physical exercise programs possibly combined with months of pharmacological treatment [48], [49]. On the other hand, obesity is associated with a defect in lipid oxidation in skeletal muscle, which may be corrected with exercise training but persists after weight loss [50]. It is possible that the small sample size and the relatively short duration of treatment may have at least in part contributed to the lack of SAT and VAT effect seen in our normal weight subjects who were instructed not to change their standard life style, diet and exercise for the entire duration of the study. Future investigations are warranted in patients, to explore substrate utilization during exercise and determine the impact of SIRT1 activation on fat volume and muscle performance capacity.

In conclusion, the present study indicates that SRT2104 was well tolerated up to 2.0 g/day for 28 days, in both elderly men and women. The highly variable pharmacokinetics observed may confound development of this particular molecule as a medicine in its current formulation, although the safety in short-term studies suggest an encouragingly high therapeutic index. This study also suggests that SRT2104 is biologically active in humans given the observed changes in some of the exploratory pharmacodynamic endpoints. This is the first study to demonstrate an impact of SRT2104 in humans on parameters that are known to lie downstream of SIRT1 activation. Given the potentially beneficial effects on serum lipids and the trends toward a beneficial effect on mitochondrial oxidative phosphorylation, the results of this study may be of use for dose selection in future clinical trials and should further stimulate interest in the testing of “second generation” sirtuin activators in adequately selected patient populations.

## Supporting Information

Methods S1 **Subject random allocation sequence and un-blinding procedures.**
(DOC)

Protocol S1 **Trial Protocol.**
(PDF)

Checklist S1 **CONSORT Checklist.**
(DOC)

Figure S1 **Study design schematic.** Subjects underwent a screening period within 21 days of the first dose. Subjects received a single daily dose of SRT2104 for 28 days and had pharmacodynamic study visits at day −1 and day 27. [* Telephone safety assessments were made approximately on days 3, 5, 10, 17, 20, 24; ** An end of study telephone safety assessment was made approximately on day 58].
(TIF)

Figure S2 **Total serum cholesterol and HDL: LDL ratios after placebo and SRT2104 treatments.** ** Denote statistical significance. Changes from baseline in total cholesterol where statistically significant at both SRT2104 doses levels on day 28 relative to baseline (0.5 g/day, p = 0.007 and 2.0 g/day, p = 0.018). Changes in HDL:LDL ratios were statistically significant (p = 0.014) in the SRT2104 2.0 g/day group on day 28 relative to baseline.
(TIFF)

Figure S3 **Time course of serum HDL and LDL cholesterol before and after 28-day treatment with SRT2104 at 0.5 g/day and 2.0 g/day doses.**
(TIFF)

Figure S4 **Individual serum cholesterol levels at baseline and after 28-day treatment with SRT2104 at 0.5 g/day and 2.0 g/day doses.**
(TIFF)

Table S1 31P ADP and PCr recovery time constant after exercise at day 27 mutations with HCC risk in the meta-analysis.
(DOC)

Table S2 Time to cessation (TTC) and rated perceived exertion (RPE) in the incremental cycle ergometer test.
(DOC)
