# Supplementary material for: A Pilot Randomized, Placebo Controlled, Double Blind Phase I Trial of the Novel SIRT1 Activator SRT2104 in Elderly Volunteers
Source: PLoS One. 2012 Dec 20;7(12):e51395. doi: 10.1371/journal.pone.0051395 (PMC3527451; doi:10.1371/journal.pone.0051395)
Supplement: Methods S1 — Subject random allocation sequence and un-blinding procedures. (DOC) [file pone.0051395.s001.doc]

**Method S1.**

**Subject random allocation sequence and un-blinding procedures.**

Sirtris Pharmaceuticals generated the subject random allocation sequence and the “Clinical Trial Material (CTM) Dispensing Order List” (containing 150 pre randomized CTM Kit numbers), which were included in the SRT-2104-007 Pharmacy Manual provided to the site. The site pharmacist assigned CTM Kit numbers to the subjects in the sequential “Dispensing Order” noted on the Pharmacy Manual to ensure that the randomization scheme was followed correctly. Screening numbers were assigned by site investigators as 3-digit numbers in sequential order starting with 001. Once it was confirmed that a subject met all eligibility criteria (including all screening laboratory test results), a 10-digit Subject ID Number was assigned in the format of: 007-042-XXXX where the first three digits corresponded to the study number (007), the second three digits reflected the site number (042), and the last four digits were the subject identifier issued by the site in numerical order, e.g. first subject enrolled = 0001, second subject enrolled = 0002, etc. Replacement subjects were assigned the same number and randomization as the subject they were replacing, however the XXXX number was prefixed with 1 (i.e. 1001 to replace 0001 etc.).

The subjects participating in the study, the study investigators, and the nursing staff at the study Centre were blind to the intervention type until the conclusion of the study. In the event the subject’s study medication had to be determined, the investigators may have broken the subject’s code by contacting the pharmacist on duty and recording the date and the reasons for breaking the blind in the CRF and in the subject’s medical records. No un-blindings were required throughout the study.
