# Supplementary material for: A Pilot Randomized, Placebo Controlled, Double Blind Phase I Trial of the Novel SIRT1 Activator SRT2104 in Elderly Volunteers
Source: PLoS One. 2012 Dec 20;7(12):e51395. doi: 10.1371/journal.pone.0051395 (PMC3527451; doi:10.1371/journal.pone.0051395)
Supplement: Protocol S1 — Trial Protocol. (PDF) [file pone.0051395.s002.pdf]

CLINICAL STUDY PROTOCOL

A Randomized, Placebo-controlled, Double-blind Clinical Study to Assess the Safety, Tolerability and Pharmacokinetics of Oral SRT2104 Capsules Administered to Healthy Elderly Subjects for 28 Days

**Protocol Number:**	SRT-2104-007
**Indication:**	N/A (non-therapeutic clinical study)
**Phase:**	I
**Design:**	Randomized, Double-Blind, Parallel, Placebo-Controlled Pharmacokinetic, Clinical Study
**Sponsor:**	Sirtris Pharmaceuticals, Inc.
**Investigational Product:**	SRT2104
**EudraCT Number:**	2009-011918-21
**Version:**	Amendment 1.2

**Protocol History**	
Original, Version 4.0	June 17, 2009
Amendment 1.1	October 9, 2009
Amendment 1.2	December 8, 2009

Sirtris Pharmaceuticals, Inc.
200 Technology Square
Cambridge, MA 02139
USA
Telephone: +1 (617) 252-6920

Approved by: Eric Jacobson, MD (signature) — 08 DEC 2009
Eric Jacobson, MD
Chief Medical Officer
Date

Confidentiality Statement
The information contained herein is confidential and the proprietary property of Sirtris Pharmaceuticals and any unauthorized use or disclosure of such information without the prior written authorization of Sirtris Pharmaceuticals is expressly prohibited.

PROTOCOL SUMMARY

Study Title: A randomized, placebo-controlled, double-blind, clinical study to assess the safety, tolerability and pharmacokinetics of oral SRT2104 capsules administered to healthy elderly subjects for 28 days

Number of Study Center(s): Single center in the United Kingdom

Study Phase: Phase I

Anticipated Study Period: Approximately 12 months

Study Objectives

Primary:

1. To determine the safety and tolerability of 28 days dosing of SRT2104 in healthy elderly subjects
2. To determine the pharmacokinetics of SRT2104 in healthy elderly subjects following single and 28 days dosing

Secondary:

1. To contrast changes in leg muscle function following repeat doses of SRT2104 or placebo:
   - Endurance exercise tolerance
   - 31P MRS measures of mitochondrial oxidative capacity in the gastrocnemius muscle
2. To test for a change in the ratio of visceral to subcutaneous body fat following repeat doses of SRT2104 relative to placebo using MRI
3. To estimate any changes in insulin sensitivity (using mOGTT) following repeat doses of SRT2104 or placebo
4. To test for dose-related effects on the exploratory pharmacodynamic measures above

Study Design: Randomized, placebo-controlled, double-blind, multiple-dose, pharmacokinetic and safety clinical study

Number of Subjects: 24 evaluable subjects

Main Criteria for Inclusion: Healthy, male and female subjects between the ages of 60-80 years of age with a BMI between18-30 kg/m^2 without any significant past or present medical condition.

Drug Supply, Dosage, and Mode of Administration: Subjects will be randomized to receive one of the following three treatments: SRT2104 0.5 g/day, SRT2104 2.0 g/day or placebo. Test material (SRT2104 and placebo) will be supplied as 250 mg capsules. Test material will be administered orally once daily for twenty-eight consecutive days. The subjects enrolled will be blinded to treatment assignment. Dosing of SRT2104 or placebo will take place at approximately the same time every morning, approximately 15 minutes following consumption of a standardized meal. Subjects must wait at least 1-2 hours after dosing before consuming additional calories. Water is permitted ad libitum.

Duration of Subject's Participation/Duration of Study/Duration of Treatment: Subject participation will include a screening period of up to 21 days, a 28-day dosing period, and a subsequent End of Dosing follow-up safety assessment 7 days following the subject's last dose of test material. Subjects will be followed for 30 days after last dose of SRT2104 to identify any possible additional adverse events.

CRITERIA FOR EVALUATION

Safety and Tolerability

The incidence of adverse events (AEs) and clinically significant abnormal laboratory values will be recorded based upon investigator observation and subject reporting. Safety will be monitored by reports of AEs (at all visits and telephone contacts after informed consent has been obtained), vital sign measurements (height [baseline only]; weight, resting pulse rate, respiration rate, temperature, and blood pressure readings), physical examinations, laboratory parameters and ECG parameters. Concomitant medications and adverse events will be recorded at every visit. Additional visits will be permitted for safety follow-up as required.

Pharmacokinetics

Plasma samples will be collected for determination of SRT2104 concentration on Days 1 and 28 at pre-dose and at 15 minutes, 30 minutes, 1 hour, 2 hours, 3 hours, 4 hours, 8 hours, and 12 hours post-dose. Plasma samples will also be collected 24 hours post-dose on Days 2 and 29.

Pharmacodynamics

Oral glucose tolerance testing will be used to assess the pharmacodynamic effects of SRT2104. At the screening visit (following informed consent) and on Day 29, blood samples will be obtained prior to the administration of 75g of oral glucose and at 10 minutes, 20 minutes, 30 minutes, 1 hour, 1.5 hours, 2 hours, and 3 hours following glucose administration.

On Day -9, an upper body structural MRI and 1H MRS will be performed to measure compartmental fat distribution. On Day -1 an exercise study of the dominant leg gastrocnemius muscle with 31P MRS will be conducted. On Day 27, the MRI, 1H MRS and 31P MRS assessments will be performed.

The MRI and 1H MRS assessments will be performed to measure compartmental fat distribution, and 31P MRS assessment will be performed on the dominant leg gastrocnemius muscle following a brief period of exercise in the MR scanner. Following the imaging procedures each subject will undergo a staged bicycle exercise test with recording of the maximum completed stage.

Biomarkers

Serum/plasma research sample(s) will be collected during this study and may be used to measure FGF-21, CRP, fructosamine, C-peptide, nicotinamide and other novel biomarkers to identify factors that may influence type 2 diabetes and/or medically related conditions, as well as the biological and clinical responses to SRT2104**.** Blood samples for exploratory transcriptomic analysis (Paxgene®) will be collected pre-dose on Days 1 and 28 and 24 hours post-dose on Days 2 and 29. A research sample for the measurement of novel biomarkers will be collected at Screening and on Days 1, 7, 14, 21 and 28. FGF-21 samples will be collected on Day 1 and 28 at pre-dose, and 1 hour, 4 hours, 8 hours post-dose and 24 hours post dose on Days 2 and 29.

General Analysis Plan

Safety evaluations will be based on the incidence, severity and type of AEs and clinically significant changes in the Subject's physical examination findings, vital signs, and clinical laboratory results. Safety variables will be tabulated and presented for all subjects who receive SRT2104 or placebo, i.e., the Safety Population. All data for SRT2104 will be grouped by dose. Data for all placebo subjects will be treated as one group. Exposure to study drug and reasons for discontinuation of study treatment will be tabulated.

Listing of individual subject and summary statistics for plasma SRT2104 concentrations, blood sampling times relative to dose, and for other pharmacokinetic parameters and graphs of concentration vs. time will be prepared by dosing cohort. Plasma concentrations and pharmacokinetic parameters will be compared between dosing cohorts using descriptive statistics. Parametric and/or non-parametric statistical contrasts between doses may be performed.

Rationale for Number of Subjects: The sample size of 24 subjects will allow for three cohorts of 8 subjects. Based on prior experience this is expected to be sufficient to estimate PK parameters for each dose.

1.1 Study Schema

Schematic Diagram of Study Design

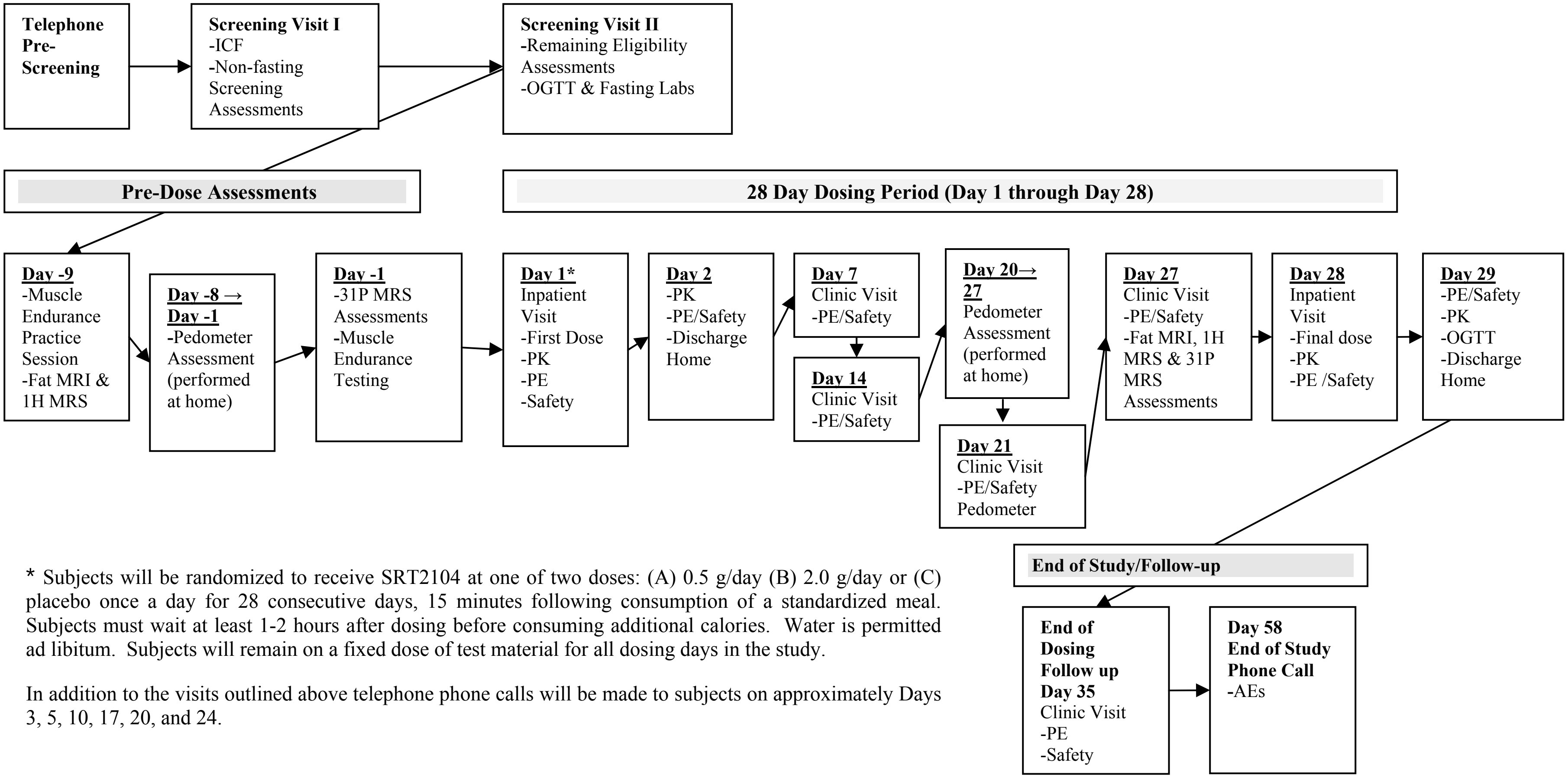

* Subjects will be randomized to receive SRT2104 at one of two doses: (A) 0.5 g/day (B) 2.0 g/day or (C) placebo once a day for 28 consecutive days, 15 minutes following consumption of a standardized meal. Subjects must wait at least 1-2 hours after dosing before consuming additional calories. Water is permitted ad libitum. Subjects will remain on a fixed dose of test material for all dosing days in the study.

In addition to the visits outlined above telephone phone calls will be made to subjects on approximately Days 3, 5, 10, 17, 20, and 24.

PE = Physical Examination
PK = Pharmacokinetic Sample Collection

1.2 Schedule of Events

Table 1: Schedule of Events

Study Period	Screening Visits I & II[25]		Pre-Dose Assessments			28 Day Dosing Period												End of Dosing Follow-up/End of Study[22]			
**Study Day**	≤ -21		-9	-8	-1	1	2	3	5	7	10	14	17	20	21	24	27	28	29	35	58
Telephone Pre-Screening[1]	X																				
Informed Consent	X																				
Medical History/ Demography/Drug & Alcohol History		X																			
Eligibility Assessment		X																			
PASE Questionnaire		X																			
Urine Drug Screen[2]		X			X												X				
Alcohol breath test		X			X												X				
BMI		X																		X	
Height		X																			
Weight		X			X	X				X		X			X		X			X	
mOGTT Testing [3]		X																	X		
Urinalysis[4]		X				X				X		X			X			X		X	
Hematology[5]		X				X				X		X			X			X		X	
Clinical Chemistry[6]		X				X				X		X			X			X		X	
Fasting Lipids[6]		X																X			
Electrolytes[7]		X				X				X		X			X			X		X	
Coagulation[8]		X				X				X		X			X			X		X	
FSH and oestradiol [9]		X																			
Vital Signs[10]		X	X		X	X	X			X		X			X		X	X	X	X	
PK[11]						X	X											X	X		
Blood sample for exploratory transcriptomic analysis (Paxgene®)[12]						X	X											X	X		
Research Blood Sample[13]		X				X				X		X			X			X			
FGF-21 Blood Sample[14]						X	X											X	X		
Physical Exam[15]		X			X	X	X			X		X			X		X	X	X	X	
12-lead ECG[16]		X			X	X	X			X		X			X		X	X	X	X	
AE/Con Med[17]		X	X		X	X	X	X	X	X	X	X	X	X	X	X	X	X	X	X	X
Fat MRI and 1H MRS Assessments[18]			X														X				

**Study Period**	**Screening Visits I & II[25]**		**Pre-Dose Assessments**			**28 Day Dosing Period**												**End of Dosing Follow-up/End of Study[24]**			
**Study Day**	**≤ -21**		**-9**	**-8**	**-1[26]**	**1**	**2**	**3**	**5**	**7**	**10**	**14**	**17**	**20**	**21**	**24**	**27[27]**	**28**	**29**	**35**	**58**
31P MRS Assessments					X												X				
MRI Safety Questionnaire		X			X												X				
Endurance Testing [19]			X		X												X				
Pedometer[20]				X	X									X	X	X	X				
Complete 10-h Overnight Fast Prior to Visit [21]		X				X	X											X	X		
Standard Meal						X	X	X	X	X	X	X	X	X	X	X	X	X			
Test Material Administrated at Site[22]						X	X											X			
Test Material Administration in Subject's Home								X	X	X	X	X	X	X	X	X	X				
Diary Card [23]						X	X	X	X	X	X	X	X	X	X	X	X	X	X	X	
Telephone Contact								X	X		X		X	X		X					X
Clinic Visit		X	X		X					X		X			X		X		X	X	
Inpatient Visit						X												X			

1. Telephone screen will be conducted prior to informed consent to gauge the subject's interest in study participation. All subjects are required to sign an informed consent form prior to undergoing any study assessments. Subjects will report to the study center to undergo the informed consent process. Fasting Screening assessments must be performed at least one day following the Informed Consent Visit.
2. Urine drug screen test will test for the following drugs of abuse: benzodiazapines, opioids, amphetamines, cocaine, and tetrahydrocannabinoids (THC)
3. mOGTT will be conducted **prior to a meal** in subjects who have fasted for at least 10 hours overnight
4. Dipstick. Microscopic exam done only if dipstick results are abnormal
5. Hematology panel includes: WBC count and differential, platelets, Hb, HCT, MCV, MCHC, MCH, RBC, RDW
6. Clinical chemistry and lipid tests include: ALT, AST, urea, serum creatinine, total bilirubin, CPK, alkaline phosphatase, lactate dehydrogenase (LDH), albumin, phosphate, uric acid, glucose, LDL, total cholesterol, HDL, triglycerides, free fatty acids
7. Electrolyte panel includes: sodium, potassium, chloride, bicarbonate, calcium, magnesium
8. Coagulation tests include: PT/INR, aPTT
9. Females only for the confirmation of menopausal status
10. Vital signs, including pulse rate, blood pressure, respiration rate, and temperature, will be collected pre-dose and 3 hours post-dose on Day 1, 2 and Day 28. Vital signs will be assessed before and after the imaging and exercise assessments on Days -1 and 27. Heart rate will be continuously monitored during the staged bicycle protocol on Days -1 and 27
11. PK samples will be collected on Days 1 and 28 at pre-dose and at 15 minutes, 30 minutes, 1 hour, 2 hours, 3 hours, 4 hours, 8 hours, 12 hours, and 24 hours post-dose on Days 2 and 29
12. Blood samples for exploratory transcriptomic analysis (Paxgene®) will be collected pre-dose on Days 1 and 28 and 24 hours post-dose on Days 2 and 29. Two Paxgene tubes will be collected at each time point
13. Research blood samples will be collected for the measurement of novel biomarkers that may include C-reactive protein, C-peptide, fructosamine, nicotinamide, and other potential biomarkers
14. FGF-21 blood samples will be collected on Day 1 and 28 at pre-dose, and 1 hour, 4 hours, 8 hours post-dose and 24 hours post-dose on Days 2 and 29
15. Complete physical exams will be conducted at Screening, Days -1, 1 , 2, 7, 14, 21, 27, 28, 29 and 35
16. 12-lead ECGs will be conducted pre-dose and 3 hours post-dose on Day 1 and Day 28. 12-lead ECGs will be done before and after the imaging and exercise assessments on Days 1- and 27. Continuous 3-lead ECG monitoring will be measured during the staged bicycle protocol on days -1 and 27. Abnormal, clinically significant ECGs should be repeated twice, at the discretion of the Investigator, to confirm the clinically significant abnormality for a total of 3 tracings. Each tracing should be obtained at least one minute apart with the subject in the supine position. QTcF values of ≥ 430msec for males and ≥450msec

for females obtained after the first dose of study drug should also be taken in triplicate, at least one minute apart with the subject in the supine position. QTcF values of ≥ 430msec for males and ≥ 450msec for females may be repeated at anytime during the study at the discretion of the investigator. Refer to Section 5.2.5 for QTc withdrawal criteria

17. Adverse events occurring prior to first dose will be recorded on the Medical History CRFs. Adverse events occurring after the first dose will be recorded on the Adverse Event CRFs. Adverse events will be followed for 30 days following test material
18. MR Imaging assessments including structural MRI and 1H MRS scan of the upper body.
19. Subjects will undergo a practice endurance (staged bicycle assessments) training session on Day -9
20. Pedometer to be worn by subjects while awake for 7 consecutive days from Day -8 and 7 consecutive days from Day 20
21. Subjects will complete a 10-h overnight fast beginning prior to the Screening OGTT and fasting laboratory assessments and before the visits on Days 1, 2 , 28, and 29
22. Test material administration (SRT2104 or placebo) will occur on all days during the study, i.e., from Day 1 to Day 28 inclusive. Subjects will take SRT2104 or placebo approximately 15 minutes following consumption of a standardized meal on all dosing days. Subjects must wait at least 1-2 hours after dosing before consuming additional calories. Water is permitted ad libitum
23. The diary card will be used to record test material dosing information and consumption of a standardized meal and will be reviewed by the investigator at each study visit
24. Subjects withdrawing from the study prior to the study assessments on Day 29 will undergo Day 35 safety assessments and receive a telephone call 30 days following the last dose of test material to assess for AEs and Concomitant Medications
25. Screening assessments will be conducted at a minimum of two separate clinic visits. The Informed Consent process will be performed at Screening Visit 1. Screening Visit II will be performed at least 1 day following Screening Visit 1 to allow subjects time to perform a 10-h overnight fast prior to the undergoing fasted laboratory assessments and mOGTT testing. Non-fasted screening assessments may be performed at either Screening Visit I or II.
26. Day-1 visit may take place on Day-2. In this case subjects will be asked to remove the pedometer during MRS scanning and exercise testing
27. Day 27 visit may take place on Day 26. In this case subjects will be asked to remove the pedometer during MRI/MRS scanning and exercise testing

TABLE OF CONTENTS

COVER PAGE

PROTOCOL SUMMARY 2
1.1 Study Schema 4
1.2 Schedule of Events 5

TABLE OF CONTENTS 8

LIST OF TABLES 10

LIST OF ABBREVIATIONS AND GLOSSARY OF TERMS 11

2 INTRODUCTION AND STUDY RATIONALE 15
2.1 Disease Background 15
2.2 Scientific Background 15
2.3 SRT2104 – Preclinical Background 16
2.4 SRT2104 – Clinical Experience 18

3 STUDY RATIONALE 21

4 STUDY OBJECTIVES 22
4.1 Primary Objectives 22
4.2 Secondary Objectives 22

5 INVESTIGATIONAL PLAN 23
5.1 Overall Study Design 23
5.2 Selection of Study Population 24
5.2.1 Number of Subjects 24
5.2.2 Inclusion Criteria 25
5.2.3 Exclusion Criteria 25
5.2.4 Removal of Subjects from Treatment or Assessment 26
5.2.5 QTc Withdrawal Criteria 27
5.3 Selection of Doses in the Study 27
5.4 Identity of Investigational Product 28
5.5 Treatments to be Administered 28
5.6 Method of Assigning Subjects to Treatment Groups 29
5.7 Unblinding Procedures 29
5.8 Duration of Treatment 30
5.9 Prior and Concomitant Treatment 30
5.10 Permitted Medications and Supportive Therapies 31
5.11 Treatment Compliance 31
5.12 Pharmacokinetic and Biomarker Sampling 32
5.13 Schedule of Events 33

6 STUDY PROCEDURES 34
6.1 Screening Procedures (≤ Day -21) 34
6.2 Imaging Assessments 42
6.3 Clinical Laboratory Assessments 43
6.4 Exercise Assessments 45
6.5 Modified Oral Glucose Tolerance Test 46

6.6 Biomarkers 46
6.7 Appropriateness of Measurements 47
6.8 Data Quality Assurance 48

7 ADVERSE EVENTS 49
7.1 Definitions 49
7.1.1 Adverse Event Definition 49
7.1.2 Serious Adverse Event Definition 49
7.2 Procedures for Recording and Reporting AEs and SAEs 50
7.3 Monitoring of Adverse Events and Period of Observation 53
7.4 Female Partner Pregnancy Reporting 54

8 STATISTICAL PROCEDURES 55
8.1 Randomization and Stratification 55
8.2 Populations for Analysis 55
8.3 Procedures for Handling Missing, Unused, and Spurious Data 55
8.4 Statistical Methods 56
8.4.1 Safety Analysis 56
8.4.2 Pharmacokinetic (PK) Analysis 56
8.4.3 Efficacy Analysis 57
8.5 Procedures for Reporting Deviations to Original Statistical Analysis Plan 58

9 ADMINISTRATIVE REQUIREMENTS 59
9.1 Good Clinical Practice 59
9.2 Ethical Considerations 59
9.3 Subject Information and Informed Consent 59
9.4 Subject Confidentiality 59
9.5 Protocol Compliance 60
9.6 Study Monitoring 60
9.7 On-site Audits 60
9.8 Case Report Form Completion 61
9.9 Drug Accountability 61
9.10 Premature Closure of the Study 62
9.11 Record Retention and Publication Policy 62
9.12 Liability and Insurance 63

10 USE OF INFORMATION 64

11 INVESTIGATOR AGREEMENT 65

12 REFERENCES 66
12.1 Declaration of Helsinki 66
12.2 Literature References 71

LIST OF TABLES

Table 1 Schedule of Events 5

LIST OF ABBREVIATIONS AND GLOSSARY OF TERMS

1H MRS	Proton Spectroscopy
31P MRS	Phosphorus-31 magnetic resonance spectroscopy
3 T	3 Tesla
3D	Three dimensional
AE	Adverse Event
ALT	Alanine Aminotransferase
ANOVA	Analysis Of Variance
aPTT	Activated Partial Thromboplastin Time
AST	Aspartate Aminotransferase
ATP	Adenosinetriphosphate
AUC	Area Under Concentration-time curve
$AUC_{0\text{-}t}$	Area under the time/plasma concentration curve (AUC) calculated by the trapezoidal method to the last measurable concentration following administration
$AUC_{o\ \tau}$	Area under the time/plasma concentration curve (AUC) calculated by the trapezoidal method to the last measurable concentration
$AUC_0\text{-}\infty$	Area under the time/plasma concentration curve (AUC) calculated by linear trapezoidal rule to the last measurable plasma concentration (Cp) with additional area calculated from Cp/Kel
BLQ	Below the Level of Quantification
BMI	Body Mass Index
BUN	Blood Urea Nitrogen
˚C	Degrees Celsius
CIC	GlaxoSmithKline Clinical Imaging Centre
C_{max}	Maximum observed Concentration
CNS	Central Nervous System
Con Med	Concomitant Medication
CPK	Creatine Phosphokinase
CRF	Case Report Form

CRO	Contract Research Organization
CRP	C-Reactive Protein
CTC	Common Toxicity Criteria
CTM	Clinical Trial Material
CV%	Coefficient of Variation (%)
CVS	Cardiovascular System
CYP	Cytochrome P450
DIO	Diet-Induced Obesity
dL	DeciLitre
ECG	Electrocardiogram
EudraCT	European Union Drug Regulating Authorities Clinical Trials
FDA	Food and Drug Administration (US)
FSH	Follicle-stimulating hormone
g	Gram
GLUT4	Glucose Transporter 4
GCP	Good Clinical Practice
Hb	Haemoglobin
HbA1c	Glycosylated Hemoglobin A
HCT	Hematocrit
HDACs	Histone Deacteylases
HDL	High-Density Lipoprotein
HIV	Human Immunodeficiency Virus
hrs	Hours
ICH	International Conference on Harmonization
IEC	Independent Ethics Committee
IMPD	Investigational Medicinal Product Dossier
IRB	Institutional Review Board
k_{el}	Terminal Elimination Rate Constant
kg	Kilogram
Km	Michaelis-Menten constant
LDH	Lactate Dehydrogenase

LDL	Low-Density Lipoprotein
LLQ	Lower Limit of Quantification
m^2	Meter Squared
MCH	Mean Corpuscular Hemoglobin
MCHC	Mean Corpuscular Hemoglobin Concentration
MCV	Mean Corpuscular Volume
MedDRA	Medical Dictionary for Regulatory Activities
mg	Milligram
µg	Microgram
MHRA	Medicines and Healthcare products Regulatory Agency
min	Minute
mIU	Milli International Units
mL	Millilitre
MR	Magnetic Resonance
MRI	Magnetic Resonance Imaging
MRS	Magnetic Resonance Spectroscopy
mmHg	Millimetres of Mercury
NCI	National Cancer Institute
NOAEL	No Observable Adverse Effect Level
ob/ob	Genetically obese mouse model, $Lep^{ob/ob}$
mOGTT	Modified Oral Glucose Tolerance Test
PASE	Physical Activity Scale in the Elderly
PCr	Phosphocreatine
PD	Pharmacodynamics
PE	Physical Examination
pg	Picogram
PGC-1α	PPAR Gamma Coactivator-1α
pH	Concentration of hydrogen ions
Pi	Inorganic Phosphate
PI	Principal Investigator
PK	Pharmacokinetics

PT/INR	Prothrombin Time/International Normalized Ratio
RBC	Red Blood Cell
RDW	Red Cell Distribution Width
REC	Research Ethics Committee
RPE	Rating of Perceived Exertion
SAE	Serious Adverse Event
SD	Standard Deviation
SIRT	Silent Information Regulator Transcript; Sirtuin
SIRT1	Sirtuin Enzyme 1
SJMC	Sir John McMichael Clinical Research Centre
SRT2104	A SIRT1 Activator
T1 w	T1 Weighted Imaging
t½	Terminal Elimination Half Life
THC	Tetrahydrocannabinoids
t_{max}	The Time of Maximum Plasma Concentration
TMF	Trial Master File
ULN	Upper Limit of Normal
WBC	White Blood Cell
WMA	World Medical Association
≤	Less Than or Equal To
≥	Greater Than or Equal To
~	Approximately

2 INTRODUCTION AND STUDY RATIONALE

2.1 Disease Background

Aging and reduced androgen levels are associated with loss of muscle mass, decreased exercise endurance and decreased muscle mitochondrial oxidative capacity even in healthy elderly people. These factors contribute to disability and dependency in later life. Problems are exacerbated by increasing immobility particularly that associated with hospitalization.

Effects of aging on muscle may show relative specificity for some muscle groups. Reductions of oxidative capacity with aging in sedentary individuals are greater in the gastrocnemius muscle than in the vastus lateralis (JA Houmard et al, 1998). This has particular clinical relevance because reductions in plantar strength and endurance in the elderly can be related independently to increased incidence of falls (GS Sorock et al, 1988).

Current approaches to management of loss of muscle power and endurance in old age are limited. Androgen treatment increases lean tissue mass and muscle strength (ET Schroeder et al, 2002), although the benefits of androgen treatment in the absence of a training intervention may be limited (ET Schroeder et al, 2003). However, potential health risks associated with long-term treatment are poorly defined and remain a concern. In women androgens can induce undesirable cosmetic features. Resistance training in the elderly can increase muscle power and bulk, reduce falls, and increase quality of life, but this demands active participation in the context of a sustained training programme.

Aerobic training increases exercise endurance and balance and may give similar benefits. Endurance exercise partly normalises insulin sensitivity and mitochondrial dysfunction in older subjects (IR Lanza et al, 2008). Home-based walking programmes are potentially practical for prescription in the community and can provide benefits associated with formal training regimes (G Baker et al, 2008; MA Tully et al, 2007). However, these demand subject motivation and may demand an interventional framework to be most effective generally.

2.2 Scientific Background

Molecular mechanisms for beneficial effects of exercise include early activation and increased transcriptional factor PGC-1α expression. The changes stimulate multiple pathways, including those

leading to increased muscle GLUT4 expression and mitochondrial biogenesis, Type I fibre differentiation, and resistance to fatigue with sustained aerobic exercise.

The potential for safer pharmacological approaches to increases in muscle endurance and enhanced oxidative metabolism has been suggested by metabolic benefits that accrue from activation of SIRT1 using resveratrol in preclinical models (T Murase et al, 2008). SIRT1 activation increases muscle PGC-1α activity. This independently provides a plausible mechanism for the increases in muscle mitochondrial biogenesis and oxidative capacity observed with resveratrol treatment (M Lagouge et al, 2006). Additional mechanisms also may contribute to reduction of insulin sensitivity. Importantly for potential therapeutic applications, SIRT1 activation using resveratrol is not associated with cardiac PGC-1α activation in rodents, and treated animals do not show signs of the cardiomyopathy associated with PGC-1α overexpression in transgenic animals (M Lagouge et al, 2006).

Phosphorus-31 magnetic resonance spectroscopy (31P MRS) is a noninvasive tool for monitoring the high-energy phosphate metabolism of skeletal muscle during exercise and recovery (PM Matthews et al, 1991, DE Larson-Meyer et al, 2000, MF Schocke et al, 2004). Tissue concentrations for phosphocreatine (PCr), adenosinetriphosphate (ATP), inorganic phosphate (Pi), and pH measured by 31P MRS are comparable to tissue obtained by invasive biochemical analysis of skeletal muscle after biopsy (DE Larson-Meyer et al, 2001). As such, 31P MRS can provide useful quantitative information of skeletal muscle oxidative capacity and mitochondrial ATP production *in vivo*.

2.3 SRT2104 – Preclinical Background

SRT2104 is a potent and selective small molecule activator of SIRT1. SRT2104 was identified as a SIRT1 activator in a high throughput screen of a diverse library of 290,000 compounds (JC Milne et al, 2007). SIRT1 activity is increased by SRT2104 due to a lowering of the K_m for its acetylated protein substrate, resulting in an approximately two-fold increase in activity. SRT2104 is selective for SIRT1 activation over the two most closely related Sirtuin homologs, SIRT2 and SIRT3. The fold selectivity for SRT2104 as compared to SIRT2 is >100 and to SIRT3 is >100.

SRT2104 was tested *in vitro* and showed no significant inhibitory activity against 39 common receptors. SRT2104 was not an inhibitor of five major cytochrome P450 isoforms tested (CYP1A,

CYP2C9, CYP2C19, CYP2D6 and CYP3A4), and is not a significant inducer of cytochrome P450 isoforms CYP1A and CYP3A4 at the concentrations tested.

The effect of once daily oral administration of SRT2104 on fasting blood glucose and fed insulin levels, body weight, triglyceride and plasma lipid levels was evaluated in a number of animal models of diabetes and obesity (DIO mice and *ob/ob* mice). In general, SRT2104 lowered fasting blood glucose and fed insulin towards normal levels and enhanced the response to a glucose tolerance test. No effect on body weight was observed with SRT2104. For more information, please refer to the SRT2104 Investigator's Brochure.

SRT2104 has demonstrated improved glucose and insulin homeostasis in Diet Induced Obese mice (DIO) and *ob/ob* mice. SRT2104 reduces glucose and insulin levels in both DIO and *ob/ob* mice after once daily oral administration at 0.1 g/kg for 7 days or more. This effect is dose-dependent and not accompanied by weight gain. The lowest dose that drives efficacy in these models corresponds to the 0.5 g/day in man when comparing blood AUC values. No adverse reactions have been seen after administration of SRT2104 in mouse models of diabetes up to a dose of 0.3 g/kg given orally once daily for 28 days.

The non-clinical safety of SRT2104 was investigated in the AMES and mouse micronucleus genetic toxicology models, and in safety pharmacology studies in rats and dogs. SRT2104 was not genotoxic and no central nervous system (CNS), cardiovascular system (CVS) or pulmonary effects were observed in the acute safety pharmacology studies at the doses up to 2.0 g/kg.

SRT2104 has been dosed up to 2.0 g/kg in 2 species (rat and dog) for 28 days. The compound was well tolerated at all doses in both species. Toxicity studies showed a NOAEL level of 2.0 g/kg in male rats and 1.0 g/kg in female rats and 1.0 g/kg in male and female dogs. In the male rat, the NOAEL was 2.0 g/kg, the highest dose tested. In the female rat, the NOAEL was considered to be 1.0 g/kg/day due to vacuolation of pancreatic acinar cells in 3/10 animals on Day 29 which was not seen after 4 weeks in the recovery animals. The physiological significance of this finding is unclear. Furthermore, this effect was not seen in the dog, even at 2.0 g/kg for 28 days. The NOAEL in the dog was determined to be 1.0 g/kg/day due to a mild increase in indirect bilirubin in the 2.0 g/kg/day group. A

significant (< 2X) increase occurred only in females in the 2.0 g/kg/day group and was not associated with changes in total bilirubin or liver enzymes.

2.4 SRT2104 – Clinical Experience

The first administration of SRT2104 to human volunteers between the ages of 18 and 55 was performed as part of the randomized, placebo-controlled, single-blind, multiple-dose, dose-escalation clinical study SRT-2104-001 (EudraCT number: 07-007598-22). A total of 28 volunteers received SRT2104 in the form of a liquid suspension as both a single dose and during a separate multiple dose period (once per day for 7 days) to healthy male volunteers at the following dose levels: 0.03, 0.1, 0.25, 0.5, 1.0, 2.0 and 3.0 g/day (4 volunteers at each dose level).

SRT2104 was well-tolerated following both the single and multiple dose periods at all dose levels investigated and all adverse events recorded were either mild or moderate in severity and were predominantly gastro-intestinal in nature. Treatment emergent adverse events observed in more than one subject were flatulence, headache, nausea, and diarrhea. All adverse events were short in duration and resolved without sequelae. No dose-related adverse events were identified.

The pharmacokinetic parameters $AUC_{(0-t)}$ and C_{max} exhibited dose proportionality over the dose range of 0.03 to 1.0 g/day in healthy volunteers. At doses greater than 1.0 g/day increases in $AUC_{(0-t)}$ and C_{max} were less than proportional to the increase in dose. The terminal elimination half-life (t½) ranged from 8 to 24 hours and was not affected by dose. There was no evidence to suggest saturation of any elimination pathways over the dose range investigated (0.03 – 3.0 g/day).

A second administration of SRT2104 to human volunteers between the ages of 18 and 65 was performed as part of a single center, combined IV and oral dosing study to evaluate the PK and absolute bioavailability of SRT2104 (Clinical Protocol SRT-2104-002; EudraCT number 2008-006283-10). SRT2104 was administered as a 250 mg oral suspension and intravenous microdose of 100µg ^{14}C-SRT2104 to eight healthy male subjects.

SRT2104 was well-tolerated by all subjects. No serious adverse events were recorded. The adverse events assessed as related to SRT2104 were aching at infusion site and parasthesia. All adverse events

were of mild severity and resolved without sequelae. The pharmacokinetic sample analysis is underway. Upon completion of PK analysis, results will be summarized in a Clinical Study Report.

A third clinical study to assess the effect of food and gender on the pharmacokinetics of SRT2104 has recently been fully enrolled (Clinical Protocol SRT-2104-004; EudraCT number: 2008-007364-41). This Phase I, randomized, open-label study enrolled 10 male healthy volunteers and 10 female healthy volunteers of non-childbearing potential between the ages of 18 and 55 to characterize the PK profile of a single 500 mg dose of SRT2104 when administered as an oral suspension and as a capsule formulation in both the fed and fasted states. In addition, safety and tolerance of SRT2104 was assessed. The clinical study report is under development.

The fourth SRT2104 study (Clinical Protocol SRT-2104-009; EudraCT number: 2009-010829-39) is a prospective, single center, clinical study of SRT2104 administered orally. This Phase 1 study is a randomized, inpatient/outpatient study to assess the safety and pharmacokinetics (PK) of SRT2104 in healthy male volunteers. Ten (10) male subjects, aged 18-60 were enrolled in this study. Subjects were randomized to receive 2.0 g SRT2104 or placebo capsules under fed conditions on eight occasions during the study; once as a single dose and once per day for seven consecutive days. The clinical portion of the study was completed and the clinical study report is under development.

The fifth SRT2104 study (Clinical Protocol SRT-2104-005; EudraCT number: 2009-010720-26) is a prospective, multi-center, clinical study of SRT2104 administered orally once daily for 28 consecutive days. The trial is a randomized, placebo-controlled, double-blind, multiple-dose, inpatient/outpatient study to assess the safety and pharmacokinetics (PK) of SRT2104 in type 2 diabetic male and female subjects on an existing, stable, background metformin therapy. Approximately 225 subjects aged 30-70, who fulfill the inclusion/exclusion criteria, will be enrolled in this study to ensure completion of forty (40) evaluable subjects within each of five dosing groups. Subjects will be evenly randomized to receive SRT2104 at one of five doses, placebo (A), 0.25 g/day (B), 0.5 g/day (C), 1.0 g/day (D), or 2.0 g/day (E), once a day for 28 consecutive days, approximately 15 minutes following consumption of a standardized meal. Subjects will remain on a fixed dose of test material for all dosing days in the study. This study is currently in development and enrollment is expected to begin in the fall of 2009.

To date (June 17, 2009), a total of 67 subjects have been dosed with SRT2104. Based on currently available clinical data, the investigational product appeared well-tolerated and no safety concerns have been identified with the administration of SRT2104 in healthy volunteers at doses up to 3.0 g per day for 7 consecutive days in the fasted state.

3 STUDY RATIONALE

SRT2104 is a small molecule SIRT1 activator that shows greater oral bioavailabity and potency than resveratrol. It has been well tolerated, in pre-clinical and clinical studies performed to date. SRT2104 offers a promising potential pharmacologic approach to increasing skeletal muscle oxidative capacity and endurance in elderly subjects. Non-clinical evidence suggests that SRT2104 may also increase insulin sensitivity, both from primary changes in muscle metabolism and secondary to other systemic metabolic changes associated with reduction of visceral body fat.

In this study, we will assess the safety, tolerability and PK of SRT2104 administered as capsules in healthy elderly subjects. Additionally, we will make a preliminary evaluation of the potential for SRT2104 to enhance muscle endurance. Two dose levels of SRT2104 will be studied.

For exploratory evaluation of pharmacodynamic measures relevant to exercise endurance, incremental cycle ergometer tests will be performed and 31P MRS will be measured to estimate changes in skeletal muscle oxidative capacity with SRT2104 administration. In addition, oral glucose tolerance test results and MRI and 1H MRS measures of body fat distribution will be evaluated as markers of metabolic responses to SRT2104.

This study, therefore, will provide PK, safety information, and exploratory pharmacodynamic data in elderly subjects to inform a decision regarding further clinical development of SRT2104 or related molecules to enhance skeletal muscle function with aging.

4 STUDY OBJECTIVES

4.1 Primary Objectives

The primary objectives of this study are:

1. To determine the safety and tolerability of 28 days dosing of SRT2104 in healthy elderly subjects
2. To determine the pharmacokinetics of SRT2104 in healthy elderly subjects following single and 28 days dosing

4.2 Secondary Objectives

The secondary objectives of this study are:

1. To contrast changes in leg muscle function following 28 days of consecutive dosing with SRT2104 or placebo:
   - Endurance exercise tolerance
   - 31P MRS measures of mitochondrial oxidative capacity in the gastrocnemius muscle
2. To test for a change in the ratio of visceral to subcutaneous body fat following repeat doses of SRT2104 relative to placebo using MRI
3. To estimate any changes in insulin sensitivity (using mOGTT) following repeat doses of SRT2104 or placebo
4. To test for dose-related effects on the exploratory pharmacodynamic measures above

5 INVESTIGATIONAL PLAN

5.1 Overall Study Design

This is a double-blind, randomized, parallel group, placebo-controlled study of healthy 60-80 year old community-dwelling male and female subjects. The study consists of approximately 9 outpatient clinic visits, 2 inpatient clinic visits and 8 telephone contacts (including the pre-screening call to determine a subject's interest in participation).

The study will be performed at the Imperial College Sir John McMichael Clinical Research Centre (SJMC), supported by the GlaxoSmithKline Clinical Imaging Centre (CIC). Both the CIC and SJMC unit are based at the Hammersmith Hospital site, London, UK.

Subjects will undergo a preliminary telephone pre-screening assessment to determine their interest in participating in the study. Once interest has been confirmed, subjects will sign an informed consent and undergo screening procedures. Screening assessments will be conducted at a minimum of two separate clinic visits. The Informed Consent process will be performed at Screening Visit 1. Screening Visit II will be performed at least 1 day following Screening Visit 1 to allow subjects time to perform a 10-h overnight fast prior to the undergoing fasted laboratory assessments and mOGTT testing. Non-fasted screening assessments may be performed at either Screening Visit I or II.

Subjects meeting the inclusion and exclusion criteria will be enrolled in the study and randomized to receive either 0.5g/day of SRT2104, 2.0 g/day of SRT2104 or placebo once daily for up to 28 consecutive days approximately 15 minutes following consumption of a standardized meal. Subjects must wait at least 1-2 hours after dosing before consuming additional calories. Water is permitted ad libitum. After the completion of the screening assessments and confirmation of eligibility, subjects will return to the site on Day -9 and will undergo a practice endurance testing session followed by a structural MRI and 1H MRS fat scan. In addition, on Day -9 subjects will be given a pedometer which they will be asked to wear while awake for 7 consecutive days from Day -8 and 7 consecutive days from Day 20 to estimate daily physical mobility levels in the home environment. Subjects will return to the site on Day -1 for safety assessments, 31P MRS assessments and exercise endurance tests.

Furthermore, subjects will return to site on Day -27 for structural MRI and 1H MRS fat scanning, 31P MRS assessments and exercise endurance testing.

On Days 1 and 28 subjects will be admitted overnight as inpatients. PK samples will be collected through 24 hours post-dose on Days 2 and 29.

During the dosing period safety visits will be performed on approximately Days 7, 14, and 21. Additional telephone safety assessments will be made approximately on Days 3, 5, 10, 17, 20 and 24.

The End of Dosing Follow-up visit will be performed approximately 35 days following the first dose of SRT2104 or placebo. An additional follow up safety telephone call will be made to each subject 30 days following their final dose of SRT2104 and/or placebo. The end of the study is defined as the last subject's last assessment (i.e. Day 58, the date of the last subject communication).

Subjects will be instructed to self administer test material at home on study days 3-27. All study visits are outpatient visits except for Day 1 and Day 28. Subjects will stay overnight at the site the evening of Day 1 and Day 28. On Day 2 and Day 29 subjects will be discharged home following the completion of study assessments and when considered clinically appropriate by the study physician.

5.2 Selection of Study Population

5.2.1 Number of Subjects

Twenty-four healthy, elderly, male and female subjects will be enrolled to provide evaluable data from 8 subjects in each of the three treatment arms.

If enrolled subjects prematurely discontinue the study, additional subjects may be enrolled as replacement subjects and assigned to the same treatment sequence at the discretion of the Sponsor in consultation with the principal investigator. However, a maximum of 12 replacement subjects will be included.

5.2.2 Inclusion Criteria

A subject will be eligible for inclusion in this study only if all of the following Screening criteria are met:

1. Independently ambulatory, healthy male and female subjects within the age range of 60 to 80 years (inclusive) at the time of screening
2. All female subjects must be of non-childbearing potential. For the purposes of this study, women need to be amenorrheic for at least 12 consecutive months, at least 6 weeks post-surgical bilateral oophorectomy (with or without hysterectomy) or post tubal ligation. Menopausal status will be confirmed by demonstrating levels of follicle stimulating hormone (FSH) 40 – 138 mIU/ml and oestradiol < 20 pg/ml at entry. In the event a subject's menopause status has been clearly established (for example, the subject indicates she has been amenorrheic for 10 years), but FSH and/or oestradiol levels are not consistent with a post-menopausal condition, determination of subject eligibility will be at the discretion of the Principal Investigator with agreement of the independent Medical Monitor
3. All male subjects must agree with their partners to use double-barrier birth control or abstinence while participating in the study and for 12 weeks following the last dose of study drug
4. Willingness to provide written informed consent to participate in the study
5. Body Mass Index (BMI) 18-30 kg/m^2 (inclusive)
6. No prior history of HIV 1 or 2
7. No prior history of disease markers for hepatitis B & C virus
8. Absence of significant disease or clinically significant abnormal laboratory values on the laboratory evaluations, medical history or physical examination during screening; normal end organ function at the discretion of the Principal Investigator
9. Have a normal 12-lead ECG or one with changes considered to be clinically insignificant on medical review. QTcF must be <430msec for males and <450msec for females
10. Resting supine BP<160/90 mmHg.
11. Comprehension of the nature and purpose of the study and compliance with the requirement of the entire protocol
12. Able to communicate in person and by telephone in a manner that allows all protocol procedures to be carried out safety and reliably in the opinion of the investigative site staff
13. Able to take 8 capsules of study medication

5.2.3 Exclusion Criteria

A subject will not be eligible for inclusion in this study if any of the following criteria are met at Screening:

1. Limitation to free passive or active movement of leg or foot by pain, prior injury, or developmental abnormality
2. Any major illness in the past three months or any ongoing chronic medical illness which in the opinion of the PI or Medical Monitor could risk subject safety or interpretation of the results

3. Ongoing or chronic history of renal or liver impairment, defined as serum creatinine clearance level less than 80ml/min as determined by Cockcroft-Gault formula (adjusted for ideal body weight), and greater than two times the upper limit of normal for liver enzymes, respectively
4. History, within 3 years, of drug abuse (including but not limited to anxiolytics or pain medication)
5. History of alcoholism (more than two years), drinkers of more than three units per day (one unit of alcohol is equivalent to one small glass of wine, half pint of beer or one measure of spirit)
6. Participation in any clinical trial with an investigative medicinal product within the past three months prior to the first dose in the current study
7. Exposure to more than three new chemical entities within 12 months prior to the first dose in the current study
8. History of difficulty in donating blood or accessibility of veins in left or right arm
9. Donation of blood (one unit or 350 ml) within three months prior to receiving the first dose of test material
10. History of sensitivity to any of the study medications, or components thereof or a history of drug or other allergy that, in the opinion of the Investigator or Medical Monitor, contraindicates their participation
11. Ongoing, or history of endocrine, inflammatory, cardiovascular (in particular cardiac hypertrophy or cardiac dysrrhythmia, cardiac failure or history of prolonged QT interval), gastro-intestinal (except for appendectomy), neurological, psychiatric or metabolic disease which in the opinion of the Investigator or Medical Monitor could risk subject safety or interpretation of the results
12. Active neoplastic disease or history of neoplastic disease (except for basal cell carcinoma of the skin)
13. Contraindications to MRI including, but not limited to: intracranial aneurism clips (except Sugita), history of metal lathe work or possibility of intra-orbital metal fragments, pacemakers and non-MR compatible heart valves or other non-MR compatible implants, history of claustrophobia or subject feels unable to lie still on their back for a period of 1 hour in the MRI scanner
14. Subjects receiving steroids, estrogens insulin or creatine will be excluded. Other concomitant medications and herbal products administered in a stable dose for at least 3 months may be permitted at the discretion of the Principal Investigator (see Section 5.9)
15. Subjects who spend less than 1 hour per week walking outside the home and subjects who participate in competitive sport with intense exercise for more than 6 hours per week
16. A positive pre-study drug/alcohol screen
17. Ongoing, or history of Type 1 or Type 2 diabetes mellitus or mOGTT results at screening that in the opinion of the PI indicates diabetes or pre-diabetes

5.2.4 Removal of Subjects from Treatment or Assessment

Subjects will be informed that they have the right to withdraw from the study at any time for any reason, without prejudice to their medical care. The investigator can withdraw subjects from the study for any of the following reasons:

- Occurrence of an unacceptable adverse event
- Subject request
- Failure to return for follow-up
- Administrative reasons
- General non-compliance with the protocol

The investigator also reserves the right to withdraw subjects in the interest of subject safety and welfare. If a subject is withdrawn from the study, they will undergo End of Dosing Follow-up/End of Study procedures. The sponsor reserves the right to terminate the study at any time for administrative reasons.

5.2.5 QTc Withdrawal Criteria

QTcF values of ≥ 430msec for males and ≥ 450msec for females may be repeated at anytime during the study at the discretion of the investigator. Abnormal, clinically significant ECGs should be repeated twice, at the discretion of the Investigator, to confirm the clinically significant abnormality for a total of 3 tracings. Each tracing should be obtained at least one minute apart with the subject in the supine position. QTcF values of ≥ 430msec for males and ≥ 450msec for females obtained after the first dose of study drug should also be taken in triplicate, at least one minute apart with the subject in the supine position.

A subject that meets the criteria below must be withdrawn from the study:

- QTcB or QTcF > 500 msec or **uncorrected** QT > 600msec (machine or manual overead)
- If subject has bundle branch block then criteria is QTcB or QTcF > 530 msec

These criteria are based on an average QTc value of triplicate ECGs. If an ECG demonstrates a prolonged QT interval, obtain two more ECGs at least one minute apart with the subject in the supine position, and then use the averaged QTc values of the three ECGs to determine whether the subject should be discontinued from the study.

5.3 Selection of Doses in the Study

The selection of the dose investigated in this study is based upon safety and pharmacokinetic data obtained from a previous clinical study involving SRT2104 (SRT-2104-001; EudraCT number: 2007-

007598-22). As part of this study SRT2104 was administered as a single acute dose and then consecutively once a day for seven days to healthy male volunteers at the following dose levels: 0.03, 0.1, 0.25, 0.5, 1.0, 2.0 and 3.0 g/day. Based on results from the SRT-2104-001 study, the SRT2104 doses to be explored in this clinical study are 0.5g/day and 2.0 g/day, orally administered once daily to subjects on all dosing days. Given the pre-clinical efficacy and toxicology data collected to date, the 0.5 g/day and 2.0 g/day doses are predicted to provide sufficient SRT2104 exposure to safely explore a potential dose-response curve.

The administration of SRT2104 with food has been shown to increase exposure levels and reduce variability in exposure levels in humans. For this reason, subjects participating in the SRT-2104-007 study are required to take SRT2104 approximately 15 minutes following the consumption of a standardized meal on each dosing day (i.e. Day 1 to Day 28). The composition of the standardized meal can be found in the Study Manual.

5.4 Identity of Investigational Product

SRT2104 drug substance is a new chemical entity which is supplied as a fine, yellowish/amber powder. The SRT2104 investigational product is prepared by packing 250 mg of the SRT2104 powder into a size 00 opaque capsule, which is then stored in dosing bottles.

For placebo product, the SRT2104 drug substance will be replaced by microcrystalline cellulose (Avicel® PH 105) to match the SRT2104 investigational product. All subjects will be provided with one dosing bottle per day that contains eight SRT2104 and/or placebo capsules for oral ingestion. The ratio of active to placebo capsules will vary according to the subject's dosing level.

A 28-day supply of test material will be packaged in a clinical trial material (CTM) kit.
Prior to being dispensed, the test material (SRT2104 and/or placebo) should be stored at 15 - 25 ^{o}C and protected from light. Subjects will be instructed to store their kits at room temperature.

5.5 Treatments to be Administered

Test material will be dispensed only to eligible subjects under the supervision of the investigator or identified sub-investigator(s). A trained investigative site member will administer the test material to

subjects on Day 1, 2, and 28. On Days 1, 2 and 28, the subjects will receive SRT2104 or placebo approximately 15 minutes following the consumption of a standard meal at the study center. Subjects must wait at least 1-2 hours after dosing before consuming additional calories. Water is permitted ad libitum. During non-clinic days, the subject will self-administer the test material approximately 15 minutes following consumption of a standard meal at home. Test material should be administered with approximately 400 mL of water at approximately the same time every dosing day (in the morning).

5.6 Method of Assigning Subjects to Treatment Groups

Subjects will be randomised 1:1:1 (0.5g/day:2.0g/day:placebo) to receive one of the following three treatments for the duration of the study:

A. 0.5g/day SRT2104 administered as two 250 mg capsules and six matching placebo capsules
B. 2.0g/day SRT2104 administered as eight 250 mg capsules
C. Matching placebo administered as eight matching placebo capsules

5.7 Unblinding Procedures

If it is medically imperative to know the dosing assignment that the subject is receiving, the Investigator shall contact the Medical Monitor to discuss the rationale for breaking the blind. The Investigator must make every attempt to contact the Medical Monitor for the study, prior to breaking the blind if the information is required for medical management of the subject. If the Investigator feels the information is urgently needed for the medical management of the subject and he/she does not have time to contact the Medical Monitor, the Investigator may break the code by contacting the pharmacist on duty at the study center.

In the event that a physician who is not associated with the study must determine the subject's study medication, the physician may break the code by contacting the pharmacist on duty at the study center. The pharmacist on duty must then notify the Investigator of the code break who will then follow the procedures outlined below.

1.1 In any scenario where the blind is broken, the following procedures must be followed:

1.1.1 The Investigator must contact the Medical Monitor within 24 hours of the code break by telephone and follow-up with a written narrative describing the event(s) that led to the code break within 48 hours of the code break.

1.1.2 If the Investigator is unable to reach the Medical Monitor and the pharmacist at the study center, then he/she may contact the designated person at Sirtris Pharmaceuticals as a last resort.

1.1.3 The Investigator must sign and date the opened unblinding envelope, as soon as is reasonably possible, but within **24 hours** of the code break, and the reason for the code break must be documented on the envelope.

1.1.4 If the blind is broken for a subject, the Investigator must record the date and the reasons for breaking the blind in the CRF and in the subject’s medical records.

The contact information for the Medical Monitor and Sirtris designee may be found in the SRT-2104-007 Pharmacy Manual.

5.8 Duration of Treatment

All subjects enrolled in the study will receive SRT2104 or placebo once daily for up to 28 days during the study period.

5.9 Prior and Concomitant Treatment

Subjects will not be allowed to participate in the study if they are receiving steroids, estrogens, insulin and creatine. Other concomitant medications and herbal products that have been administered in a stable dose for at least 3 months are permitted on this study unless in the opinion of the Principal Investigator and Medical Monitor the medication will interfere with the study procedures or compromise subject safety. Subjects should maintain a stable dose of medication throughout the duration of the trial unless the Medical Monitor considers such changes will not risk subject safety or interpretation of study data.

All medications that are administered during the study must be recorded in the subject’s CRF and in the source documents.

5.10 Permitted Medications and Supportive Therapies

Medications will be permitted as necessary to treat an adverse event occurring during the study.

5.11 Treatment Compliance

Compliance for dosing after oral administration of SRT2104 or placebo will be assessed by trained study personnel. Subjects are required to bring with them all study medication (both empty and filled bottles) and diary card to study visits. Empty bottles should be returned to the site in an ongoing basis. A subject diary card will be provided in which subjects confirm consumption of a standardized meal and record the date of dose, time of dose and number of capsules taken. A new diary card will be issued by the site staff at each weekly visit. Diary cards will be reviewed with the patient for compliance on an ongoing basis and completed diary cards will be collected by site staff on a weekly basis. Drug accountability will be performed to estimate treatment compliance.

If a subject withdraws from the study or does not comply, they may be replaced at the discretion of the Principal Investigator, Medical Monitor, and Sponsor; in that event, the subject will cease participation in the study but will still be considered evaluable for purposes of safety. If a subject does not comply with the protocol or withdraws from the study, they will undergo End of Dosing Follow-up/End of Study visit procedures after last dose of test material.

Subjects who miss either three consecutive doses or six individual doses of test material at any time point during the study will be automatically withdrawn from the study. If a subject does not comply with the protocol or withdraws from the study, they will undergo Day 35 End of Dosing/End of Study procedures after last dose of test material.

Subjects are expected to take SRT2104 or placebo approximately 15 minutes following the consumption of a standard meal and wait at least 1-2 hours after dosing before consuming additional calories on all 28 dosing days. Water is permitted ad libitum. Subjects are expected to refrain from excessive alcohol use while on the study. Caffeine is permitted in moderation during the study however subjects must refrain from caffeine and alcohol for 24 hours prior to screening and imaging

assessments. Activity levels should remain constant wherever possible throughout the duration of the study. Initiating intense exercise or physical activity should be discussed with a physician prior to participation.

5.12 Pharmacokinetic and Biomarker Sampling

The pre-dose PK sample will be collected within one hour prior to test material administration. The post-dose PK samples will be collected within 2 minutes of the scheduled time where the end time of collection will be recorded to the nearest minute. The reason for any deviation from the scheduled collection time will be recorded promptly in the relevant source and CRF. On Day 2 and Day 29, the 24 hour post-dose assessment PK sample must be collected from subjects before standard meal consumption. On Day 2 the 24 hour PK sample must be taken prior to the Day 2 dose being administered.

PK Samples will be collected as follows during the study period:

**Day 1**	Pre-dose, and 15 minutes, 30 minutes, 1 hour, 2 hours, 3 hours, 4 hours, 8 hours and 12 hours post-dose
**Day 2**	24 hours post-Day 1 dose
**Day 28**	Pre-dose, and 15 minutes, 30 minutes, 1 hour, 2 hours, 3 hours, 4 hours, 8 hours and 12 hours post-dose
**Day 29**	24 hours post-Day 28 dose

Blood samples will be collected in pre-labelled vacutainers for PK analysis. The sample will be separated by centrifugation at 1500xg and 4°C for 15 minutes. Two equal aliquots of plasma will be transferred to polypropylene vials labelled identically to the original blood sample and stored at approximately -20°C pending further analysis. Samples will be transferred to a bioanalytical laboratory for measurement of plasma SRT2104 concentration. Samples may also be further divided and transferred to Sirtris Pharmaceuticals for the measurement of biomarkers related to research into type II diabetes.

5.13 Schedule of Events

A detailed visit-by-visit schedule of study procedures is provided in Table 1, following the protocol summary. Prior to engaging in any study procedure, each subject must sign and date an informed consent form.

6 STUDY PROCEDURES

All study procedures are reflected in the Schedule of Events (Section 1.2).

6.1 Screening Procedures (≤ Day -21)

Potential subjects initially will be telephone screened to determine potential eligibility for the study. Potential subjects who express interest in participating in the study will be sent the full patient information sheet and consent form for their review and two screening appointments will be scheduled at the site. The Informed Consent process will be performed at Screening Visit 1. Screening Visit II will be performed at least 1 day following Screening Visit 1 to allow subjects time to perform a 10-h overnight fast prior to the undergoing fasted laboratory assessments and mOGTT testing. Non-fasted screening assessments may be performed at either Screening Visit I or II. Potential subjects will be given an opportunity to have any questions about the study or their participation in it answered by the PI or his designate. Prior to engaging in any study procedure, each potential subject must sign and date an informed consent form. When the consent form is signed, each subject will be assigned a unique screening number. Subjects will be required to fast for at least 10 hours prior to their arrival at the site (in preparation for the mOGTT) for the Screening Visit II. The following procedures will be performed during the screening period after Informed Consent is obtained:

- Confirm completion of 10 hour overnight fast*
- Medical history and demographics
- History of drug and alcohol use
- Record of any prior or current medications
- A complete physical examination including vital signs (pulse rate, blood pressure, respiration rate and temperature) measured
- BMI (Height and body weight)
- 12-lead ECG. Subject should be lying comfortably in the supine position with ECG leads on for at least 10 minutes prior to ECG recording.
- Blood and urine samples will be taken for clinical laboratory assessment (hematology, clinical chemistry, electrolytes, coagulation, fasting lipid profile* and urinalysis). Confirmation of menopausal status will also be performed for all female subjects (FSH and oestradiol)
- Blood sample collection for biomarker analysis (C-reactive protein, C-peptide, fructosamine, nicotinamide and other potential biomarkers)

- Urine screen for drugs of abuse
- Alcohol breath test
- PASE activity questionnaire
- MRI safety questionnaire
- Modified Oral glucose tolerance test (mOGTT) (see Protocol Section 6.5)*
- Final evaluation of eligibility for study enrollment

*Note: Informed Consent must be obtained prior to all study assessments at Screening Visit I. Completion of the 10 hour overnight fast, mOGTT and fasting lipid profile are to be performed only at Screening Visit II only. All other assessments may be performed at either Screening Visit I or II.

Following successful screening, subjects will be randomized to receive test material.

Day -9

Those subjects meeting study criteria for continuation in the study then will return to the site on Day -9. Each subject will be given a pedometer, which they will be asked to wear at home during waking hours beginning the morning of Day -8 for 7 consecutive days. The following assessments/procedures will be performed:

- Vital signs assessment
- Review of prior and current medications
- AE assessment
- MRI safety questionnaire

Subjects will undergo a familiarization (practice) session with the exercise assessment equipment and the graded exercise testing procedure.
Subjects will then undergo the following imaging assessment

- Upper body structural MRI and 1H MRS to measure compartmental fat distribution

Day -1

The baseline evaluation visit at the site will take place within 20 days of screening. The following assessment will be performed:

- Review and record pedometer data
- Vital signs assessment

- Review of prior and current medications
- AE assessment
- 12-lead ECG. Subject should be lying comfortably in the supine position with ECG leads on for at least 10 minutes prior to ECG recording
- Weight
- Alcohol breath test
- Urine drugs screen
- Physical exam
- MRI safety questionnaire

Subjects will then undergo the following imaging assessment

- Exercise study of the dominant leg gastrocnemius muscle with 31P MRS

And after the imaging study, the following exercise assessment will be performed:

- A staged bicycle exercise protocol will then be performed with recording of the maximum completed stage
- Continuous heart rate and 3-lead ECG monitoring will be performed during the staged bicycle exercise protocol. Data from these assessments will not be entered on to the CRFs however clinically significant results will be recorded as Adverse Events
- A 12-lead ECG and vital signs will be measured prior to the test and approximately 20 minutes after completing the exercise test

It is preferable that this visit and these assessments take place on Day-1, however if this is not possible this visit may take place on Day-2. In this case subjects will be asked to remove the pedometer during MRS scanning and exercise testing.

Day 1

On Day 1 subjects will be admitted for an overnight stay. Prior to arrival subjects will have fasted for at least 10 hours.

The following assessments will be performed:

- Confirm completion of 10 hour overnight fast
- Vital signs assessment

- Review of concomitant medications
- AE assessment
- Physical exam
- Weight
- Diary card administration & instructions
- 12-lead ECG. Subject should be lying comfortably in the supine position with ECG leads on for at least 10 minutes prior to ECG recording
- Blood and urine samples will be taken for clinical laboratory assessment (clinical chemistry, coagulation, haematology, electrolytes, urinalysis)
- Blood sample collection for biomarker analysis (C-reactive protein, C-peptide, fructosamine, nicotinamide and other potential biomarkers)
- Pre-dose blood sample collection for exploratory transcriptomic analysis (Paxgene®). Two Paxgene blood collection tubes will be obtained
- Pre-dose (t=0) blood sample collection for PK assessment in pre-labelled vacutainers
- Pre-dose FGF-21 blood sample collection

Test material will be administered approximately 15 minutes following consumption of a standardized meal. Subjects must wait at least 1-2 hours after dosing before consuming additional calories. Water is permitted ad libitum. The following procedures will be performed on Day 1 **following** test material administration:

- Record dose administration
- Post-dose vital signs (pulse rate, blood pressure, respiration rate and temperature) at 3 hours post-dose measured in the supine position
- 12-lead ECG 3 hours post-dose. Subject should be lying comfortably in the supine position with ECG leads on for at least 10 minutes prior to ECG recording
- Post-dose blood sample collection for PK analysis in pre-labelled vacutainers at 15 minutes, 30 minutes, 1 hour, 2 hours, 3 hours, 4 hours, 8 hours, 12 hours post-dose
- Post-dose FGF-21 blood sample collection (1 hour, 4 hours and 8 hours)
- Adverse event assessment

Day 2

Subjects will have fasted for at least 10 hours (overnight). The following assessments will be performed:

- Confirm completion of 10 hour overnight fast
- Vital signs assessment
- Diary card review
- AE and concomitant medication review
- PK collection in pre-labelled vacutainers at 24 hours post Day 1 dose
- FGF-21 blood sample collection at 24 hours post-Day 1 dose
- Blood sample collection for exploratory transcriptomic analysis (Paxgene®) at 24 hours post-Day 1 dose. Two Paxgene blood collection tubes will be obtained

The second dose of test material will be administered approximately 15 minutes following the consumption of a standardized meal. Subjects must wait at least 1-2 hours after dosing before consuming additional calories. Water is permitted ad libitum. The following procedures will be performed on Day 2 following test material administration:

- Record dose administration
- 12-lead ECG. Subject should be lying comfortably in the supine position with ECG leads on for at least 10 minutes prior to ECG recording
- Physical examination
- Vital signs
- AE review
- Discharge when considered clinically appropriate by the study physician

Days 3, 5, 10, 17, 20, and 24

On approximately Days 3, 5, 10, 17, 20 and 24 subjects will be contacted by telephone to identify adverse events, concomitant medications, and need for short-term follow up. Subjects will be telephoned by the site staff and reminded to wear the pedometer beginning on the morning of Day 20 while awake for 7 consecutive days. All telephone contacts will be made as near as possible to the scheduled day.

Days 7, 14, and 21

On Days 3-27 subjects will self-administer test material at home approximately 15 minutes following consumption of a standardized meal. Subjects must wait at least 1-2 hours after dosing before consuming additional calories. Water is permitted ad libitum. Subjects will take the test

material at approximately the same time of day (in the morning) with ~400 mL of water. Subjects will return to the site on approximately Days 7, 14 and 21 for the following safety assessments.

- Review of concomitant medications
- Vital signs assessment
- AE assessment
- Physical exam
- Weight
- Diary card review, collection and provision of a new diary card
- 12-lead ECG. Subject should be lying comfortably in the supine position with ECG leads on for at least 10 minutes prior to ECG recording
- Blood and urine samples will be taken for clinical laboratory assessment (clinical chemistry, coagulation, haematology, electrolytes, urinalysis)
- Blood sample collection for biomarker analysis (C-reactive protein, C-peptide, fructosamine, nicotinamide and other potential biomarkers)

Theses visits will be scheduled to take place as near as possible to the scheduled day. The actual day of the visit will be recorded in the CRF.

Day 27

On Day 27 subjects will return to the site for the following assessments/procedures:

- Vital signs assessment
- Review and record pedometer data
- Physical examination
- Urine drug screen
- Alcohol breath test
- Review of concomitant medications
- AE assessment
- Diary card review
- 12-lead ECG. Subject should be lying comfortably in the supine position with ECG leads on for at least 10 minutes prior to ECG recording
- Weight
- MRI safety questionnaire

Subjects will then undergo the following imaging assessments:

- Upper body structural MRI and 1H MRS to measure compartmental fat distribution
- Exercise study of the dominant leg gastrocnemius muscle with 31P MRS

After the imaging study, the following exercise assessment will be performed:

- A staged bicycle exercise protocol will then be performed with recording of the maximum comfortable stage
- Continuous heart rate and 3-lead ECG monitoring will be performed during the staged bicycle exercise protocol. Data from these assessments will not be entered on to the CRFs however clinically significant results will be recorded as Adverse Events
- A 12-lead ECG and vital signs will be measured prior to the test approximately 20 minutes after completing the exercise test
- It is preferable that this visit and these assessments take place on Day 27, however if this is not possible this visit may take place on Day 26. In this case subjects will be asked to remove the pedometer during MRI/MRS scanning and exercise testing.

Day 28

On Day 28 subjects will be admitted to the site for an overnight stay. Subjects will arrive following an overnight fast of at least 10 hours.

The following assessments/procedures will be performed:

- Confirm completion of 10 hour overnight fast
- Vital signs assessment
- Review of concomitant medications
- AE assessment
- Physical exam
- Diary card review and collection
- 12-lead ECG. Subject should be lying comfortably in the supine position with ECG leads on for at least 10 minutes prior to ECG recording
- Blood and urine samples will be taken for clinical laboratory assessment (clinical chemistry, coagulation, haematology, electrolytes, fasting lipids and urinalysis)
- Blood sample collection for biomarker analysis (C-reactive protein, C-peptide, fructosamine, nicotinamide and other potential biomarkers)

- Pre-dose blood sample collection for exploratory transcriptomic analysis (Paxgene®). Two Paxgene blood collection tubes will be obtained
- Pre-dose (t=0) PK blood sample collection in pre-labelled vacutainers
- Pre-dose FGF-21 blood sample collection

Test material will be administered approximately 15 minutes following consumption of a standardized meal. Subjects must wait at least 1-2 hours after dosing before consuming additional calories. Water is permitted ad libitum. The following procedures will be performed on Day 28 following test material administration:

- Record dose administration
- Post-dose PK blood sample collection in pre-labelled vacutainers at 15 minutes, 30 minutes, 1 hour, 2 hours, 3 hours, 4 hours, 8 hours, 12 hours post-dose
- Post-dose FGF-21 blood sample collection (1 hour, 4 hours and 8 hours)
- Post-dose vital signs (pulse rate, blood pressure, respiration rate and temperature) at 3 hours post-dose measured in the supine position
- 12-lead ECG 3 hours post-dose. Subject should be lying comfortably in the supine position with ECG leads on for at least 10 minutes prior to ECG recording
- Adverse event assessment

Day 29

On Day 29 subjects will undergo oral glucose tolerance testing and must have completed an overnight fast of at least 10 hours. No test material is administered on Day 29. The following assessments/procedures will be performed:

- Confirm completion of 10 hour overnight fast
- Vital signs assessment
- Review of concomitant medications
- AE assessment
- Physical examination
- Post-dose PK blood sample collection in pre-labelled vacutainers 24 hours post-dose
- FGF-21 blood sample collection at 24 hours post-Day 28 dose
- Blood sample collection for exploratory transcriptomic analysis (Paxgene®) at 24 hours post-Day 28 dose. Two Paxgene blood collection tubes will be obtained
- Modified Oral glucose tolerance test (mOGTT) (see Protocol Section 6.5)

- 12-lead ECG. Subject should be lying comfortably in the supine position with ECG leads on for at least 10 minutes prior to ECG recording
- Note: The pre-glucose administration blood sample for the mOGTT test taken about 10 minutes prior to glucose administration should be collected at approximately the same time as the, 24-hour FGF-21, Paxgene and PK samples. With the exception of the PK and FGF-21 samples, each blood sample should be collected in separate blood collection tubes

Day 35

On approximately Day 35 subjects will return to the site for safety assessments:

- Review of concomitant medications
- Vital signs assessment
- AE assessment
- Physical exam
- Weight
- BMI
- 12-lead ECG. Subject should be lying comfortably in the supine position with ECG leads on for at least 10 minutes prior to ECG recording
- Blood and urine samples will be taken for clinical laboratory assessment (clinical chemistry, coagulation, haematology, electrolytes, urinalysis)

Subjects will be discharged from the study when considered appropriate by the investigator.

Day 58

Approximately thirty days following the final dose of test material subjects will be followed via telephone for the follow-up of any adverse events and concomitant medications.

6.2 Imaging Assessments

All imaging assessments will be performed on a 3T Siemens scanner MRI at the CIC. Prior to each MRI session subjects will undergo an MRI safety questionnaire and will be assessed for their suitability for scanning on that day.

Structural MRI of Quadriceps and Gastrocnemius Muscle

Initial structural MRI scans (T1w) of the leg may be acquired to provide measures of anatomy guiding coil placement and the interpretation of scans below.

Upper Body Structural MRI and 1H MRS to Measure Compartmental Fat Distribution

MRI scan sessions will consist of, but are not limited to, the acquisition of the following sequences:

- Positioning in magnet
- Rapid 3D Fat/Water scan- shoulders to pelvic floor
- Breath held single voxel 1H MRS of liver

31P MRS Evaluation of Gastrocnemius Muscle

Subjects will be positioned in the scanner such that the calf muscle of the dominant leg is in the isocentre of the magnet. A 31P MRS surface coil will be applied to the calf surface over the gastocnemius muscle belly. Subjects will be asked to sustain a few minutes of foot movement and then to relax the leg and foot. 31P MRS data will be acquired continuously during movement and in the post-exercise metabolic recovery phase. Data thus will be acquired reflecting muscle intracellular pH and phosphate metabolite concentration changes with exercise and recovery from which the rate of muscle mitochondrial oxidative phosphorylation can be estimated.

6.3 Clinical Laboratory Assessments

Hematology

White blood cell (WBC) count	Complete WBC differential
Hemoglobin	Platelets
Hematocrit	Mean corpuscular volume (MCV)
Mean Corpuscular Hemoglobin Concentration (MCHC)	Red blood cell (RBC) count
	RDW
Mean Corpuscular Hemoglobin (MCH)	

Clinical Chemistry, Coagulation and Electrolytes

Sodium	Alanine aminotransferase (ALT)
Potassium	Aspartate aminotransferase (AST)
Chloride	Total, direct, and indirect bilirubin
Urea	Alkaline phosphatase
Serum creatinine	Lactate dehydrogenase (LDH)
Glucose	Bicarbonate
Calcium	Albumin
Magnesium	Phosphate
Uric acid	Creatine Kinase
PT/INR	aPTT

Menopausal/pregnancy status

Follicle stimulating hormone (FSH)

Oestradiol

Urinalysis

Dipstick	Microscopic exam only if dipstick abnormal

Lipid Profile

Total Cholesterol	LDL
HDL	Triglycerides
Free Fatty Acids	

Biomarkers

Nicotinamide	CRP
Fructosamine	FGF-21
C-peptide	

mOGTT

Glucose	C-peptide
Insulin	

Drugs of Abuse (urine)

Benzodiazapines	Opioids
Amphetamines	Cocaine
Tetrahydrocannabinoids (THC)	

6.4 Exercise Assessments

Testing will be conducted at room temperature and (whenever possible) at a consistent time for each subject.

Incremental Cycle Ergometer Exercise Test

After resting for 10 minutes participants will undergo an incremental exercise test on a cycle ergometer. The test will consists of 2 minute stages after an initial 3 minutes unloaded cycling (ACSM (2005) American College of Sports Medicine). Work load will be progressed in staged increments from unloaded cycling. The test will be ended at volitional exhaustion or if the participant is unable to maintain the required 50 rpm cadence. Heart rate, 3-lead ECG, cycle cadence and work rate and perceived exertion (RPE scale (Borg, G. (1998) Borg's perceived exertion and pain scales. Champaign, IL) will be monitored throughout the test. Individuals will be instructed to cycle until they are unable to continue. Subjects will be encouraged to make a maximal comfortable effort in the exercise test. Standardised encouragement and instructions will be given to all participants.

6.5 Modified Oral Glucose Tolerance Test

In this study a mOGTT will be used to assess the PD effects of SRT2104. After the ingestion of the 75g oral glucose challenge, there is a rapid rise in insulin secretion (first phase response), which is followed by a more sustained release of the hormone (second phase). During this secretion process, C-peptide, or connecting peptide, is split from pro-insulin, the insulin precursor molecule, and is produced in equimolar amounts to insulin. In the bloodstream, C-peptide has a long half-life, because, unlike insulin, it is not subject to hepatic clearance. The first and second phases of insulin secretion are derived by modeling the C-peptide and insulin kinetics data during the mOGTT. In addition, insulin sensitivity can be calculated from the rate of appearance and disappearance of the ingested glucose.

After an overnight fast, the subject will drink the standard glucose beverage containing 75g of glucose within 15 minutes. A blood sample will be drawn 10min before and just prior to the subject consuming the beverage. Additional blood samples will be drawn at 10, 20, 30, 60, 90, 120 and 180 minutes after the subject has consumed the glucose beverage. These blood samples will be used to estimate any changes in glucose and insulin levels.

6.6 Biomarkers

With the subject's consent, plasma/serum research sample(s) will be collected during this study and will be used for the purposes of measuring CRP, fructosamine, nicotinamide and other novel biomarkers to identify factors that may influence type 2 diabetes and/or medically related conditions, as well as the biological and clinical responses to SRT2104. If relevant, this approach will be extended to include the identification of biomarkers associated with adverse events. Additionally, blood samples for exploratory transcriptomic analysis using Paxgene tubes will also be collected. Two Paxgene blood collection tubes will be obtained at each sampling time point to ensure there is enough material to perform the exploratory transcriptomic analysis.

Research samples for exploratory transcriptomic analysis using Paxgene tubes will be collected twice during the active treatment phase of the study; at the beginning of the study (Day 1 and Day 2) and at the end of the study (Day 28 and Day 29). These samples will be collected pre-dose on

Days 1 and 28 and 24 hours post-dose on Days 2 and 29. The Paxgene samples from subjects randomized to the placebo arm will not be analyzed by the laboratory.

In addition, FGF-21 samples will be collected at approximately the same time the PK samples are obtained. The plasma/serum research samples collection times and dates will be limited to Day 1 at pre-dose 1 hour, 4 hours, 8 hours, and 24 hours post-dose and Day 28 at pre-dose, 1 hour, 4 hours, 8 hours, and 24 hours post-dose.

A research sample for the measurement of novel biomarkers that may include C-reactive protein, C-peptide, fructosamine, nicotinamide, or other potential biomarkers will be collected at Screening, and on Days 1, 7, 14, 21 and 28.

Samples will be collected at the time points indicated in the Schedule of Events (Table 1). The timing of the collections may be adjusted on the basis of emerging PK or PD data from this study or other new information in order to ensure optimal evaluation of the PD time points.

All samples will be retained for a maximum of 15 years after the last subject completes the trial.

6.7 Appropriateness of Measurements

Per the US Food and Drug Administration (FDA) guidance, "Collection of Race and Ethnicity Data in Clinical Trials, September 2005", demographic data and complete subject medical histories will be documented for all subjects during screening. Study drug administration data, including dose interruptions and modifications and the associated reason(s), also will be documented.

AEs and SAEs will be monitored in this study in accordance with International Conference on Harmonization (ICH) Good Clinical Practice (GCP) guidelines to ensure the safety of subjects.

6.8 Data Quality Assurance

Sirtris or its designated representative will conduct a clinical site visit to verify the qualifications of the investigator, inspect clinical site facilities, and inform the Investigator of responsibilities and procedures for ensuring adequate and correct study documentation.

The Investigator is required to prepare and maintain adequate and accurate case histories designed to record all observations and other data pertinent to the study for each study participant. All information recorded on the CRFs for this study must be consistent with the subject's source documentation.

All study data recorded on source documents will be entered into the CRFs.

During the course of the study, the study monitor will conduct clinical site visits to review protocol compliance, compare CRFs and individual subject's medical records (source documents), assess drug accountability, and ensure that the study is being conducted according to pertinent regulatory requirements. CRFs will be verified with source documentation. The review of medical records will be performed in a manner to ensure that subject confidentiality is maintained. A hardcopy of the final CRFs will be placed in the Investigator's study file and Sirtris' Trial Master File.

Instances of missing or uninterpretable data will be discussed with the Investigator for resolution. Study data will be entered into a secure, validated data processing system and a backup will be maintained. Any changes to study data will be documented. A quality assurance audit will be performed on the database.

7 ADVERSE EVENTS

The principal investigator and designated study staff are responsible for detecting, documenting and reporting events that meet the definition of an AE or SAE.

7.1 Definitions

7.1.1 Adverse Event Definition

An **adverse event** (AE) is any untoward medical occurrence in a subject administered a pharmaceutical product, which does not necessarily have a causal relationship with the treatment. An AE can be any unfavorable and unintended sign (e.g., including an abnormal laboratory finding), symptom, or disease temporally associated with the use of the study drug, whether or not it is considered to be study drug-related. This includes any newly occurring event or previous condition that has increased in severity or frequency since the administration of study drug.

7.1.2 Serious Adverse Event Definition

A **serious adverse event** (SAE) is any AE, occurring at any dose and regardless of causality that:

- Results in **death**.
- Is **life-threatening**. Life-threatening means that the subject was at immediate risk of death from the reaction as it occurred, i.e., it does not include a reaction which hypothetically might have caused death had it occurred in a more severe form.
- Requires inpatient **hospitalization or prolongation of existing hospitalization**. Hospitalization admissions and/or surgical operations scheduled to occur during the study period, but planned prior to study entry, are not considered AEs if the illness or disease existed before the subject was enrolled in the trial, provided that it did not deteriorate in an unexpected manner during the study (e.g., surgery performed earlier than planned).
- Results in **persistent or significant disability/incapacity**. Disability is defined as a substantial disruption of a person’s ability to conduct normal life functions.
- Is a **congenital anomaly/birth defect**.

- Is an **important medical event**. An important medical event is an event that may not result in death, be life-threatening, or require hospitalization but may be considered an SAE when, based upon appropriate medical judgment, it may jeopardize the subject and may require medical or surgical intervention to prevent one of the outcomes listed in the definitions for SAEs. Examples of such medical events include allergic bronchospasm requiring intensive treatment in an emergency room or at home, blood dyscrasias or convulsions that do not result in inpatient hospitalization, or the development of drug dependency or drug abuse.

Clarification should be made between the terms “serious” and “severe” since they ARE NOT synonymous. The term “severe” is often used to describe the intensity (severity) of a specific event (as in mild, moderate, or severe myocardial infarction); the event itself, however, may be of relatively minor medical significance (such as a severe headache). This is NOT the same as “serious,” which is based on subject/event outcome or action criteria described above, and is usually associated with events that pose a threat to a subject’s life or functioning. A severe AE does not necessarily need to be considered serious. For example, persistent nausea of several hours’ duration may be considered severe nausea but not an SAE. On the other hand, a stroke resulting in only a minor degree of disability may be considered mild, but would be defined as an SAE based on the above noted criteria. Seriousness (not severity) serves as a guide for defining regulatory reporting obligations.

7.2 Procedures for Recording and Reporting AEs and SAEs

All AEs spontaneously reported by the subject and/or in response to an open question from study personnel or revealed by observation, physical examination or other diagnostic procedures will be recorded on the appropriate page of the CRF. Any clinically relevant deterioration in laboratory assessments or other clinical findings is considered an AE and must be recorded on the appropriate pages of the CRF. When possible, signs and symptoms indicating a common underlying pathology should be noted as one comprehensive event.

All SAEs that occur during the course of the study, as defined by the protocol, must be reported by the investigator to Sirtris and Akos Ltd by faxing the SAE form **within 1 working day** from the point in time when the investigator becomes aware of the SAE. In addition all SAEs, including all

deaths, which occur up to and including 30 days after administration of the last dose of study drug, must be reported to Sirtris within 1 working day. All SAEs and deaths must be reported whether or not considered causally related to the study drug. The information collected using the SAE form will include a minimum of the following: Subject number, a narrative description of the event and an assessment by the investigator as to the intensity of the event and relatedness to study drug. A sample of the SAE form can be found in the study manual. Follow-up information on the SAE may be requested by Sirtris.

SAE Reporting Contact Information:

Maria Hilling
Drug Safety/Pharmacovigilance Vice President
Akos Ltd., The Coach House, Pipers Lane, Harpenden AL5 1AH UK

SAE Reporting Line: +44 1582 761888
Telephone: +44 1582 766339

And to the Sponsor:

Eric Jacobson, MD
Chief Medical Officer

Direct Line - +1 617-252-6920 x2208
Fax Line - +1 617-679-8499

Sirtris Pharmaceuticals
200 Technology Square
Cambridge, MA 02139
USA

If there are serious, unexpected adverse drug reactions associated with the use of the study drug, Akos Ltd. will notify the appropriate regulatory agency(ies) and all participating investigators on an expedited basis (7 days for fatal or life-threatening serious, unexpected adverse drug reactions). Akos Ltd. will promptly notify the Institutional Review Board (IRB)/Independent Ethics Committee (IEC) of all unexpected serious adverse drug reactions involving risk to human subjects

in accordance with the rules and regulations of the IRB/IEC. An unexpected event is one that is not reported in the Investigator's Brochure.

The events that resulted in planned hospital admissions or surgical procedures for an illness or disease that existed before the subject was enrolled in the study or before study drug was given, are not to be considered AEs unless they cause the planned hospital admission or surgical procedure to occur at a time other than the planned date.

For both serious and non-serious AEs, the investigator must determine both the intensity of the event and the relationship of the event to study drug administration.

Intensity for each AE will be determined by using the NCI CTC (Version 4.0) as a guideline, wherever possible. Dose-limiting toxicities will be defined as those AEs of grade 3 or greater that are seen to occur with an evident temporal relationship to SRT2104 dosing. In those cases where the NCI CTC criteria do not apply, intensity should be defined according to the following criteria:

Mild	Awareness of sign or symptom, but easily tolerated
Moderate	Discomfort enough to cause interference with normal daily activities
Severe	Inability to perform normal daily activities
Life Threatening or disabling	Immediate risk of death from the reaction as it occurred
Death	The event resulted in death

Relationship to study drug administration will be determined as follows:

Not Related	No relationship between the experience and the administration of study drug; related to other etiologies, such as concomitant medications or subject's clinical state.
Unlikely Related	The current state of knowledge indicates that a relationship is unlikely.

Possibly Related	A reaction that follows a plausible temporal sequence from administration of the study drug and follows a known response pattern to the suspected study drug. The reaction might have been produced by the subject's clinical state or other modes of therapy administered to the subject.
Related	A reaction that follows a plausible temporal sequence from administration of the study drug and follows a known response pattern to the suspected study drug and can be confirmed with a positive re-challenge test or supporting laboratory data.

7.3 Monitoring of Adverse Events and Period of Observation

AEs, both serious and non-serious, and deaths will be recorded on the CRFs throughout the study from the time of consent. All SAEs should be monitored until they are resolved or are clearly determined to be due to a subject's stable or chronic condition or intercurrent illness(es). Any SAE that occurs at any time after completion of the study and the designated follow-up period, which the investigator considers to be related to study medication, must be reported to Sirtris.

AEs or SAEs requiring therapy must be treated by recognized standards of medical care to protect the health and well-being of the subject. Appropriate resuscitation equipment and medicines must be available to ensure the best possible treatment of an emergency situation.

The outcome of AEs will be rated as:

- Recovered/Resolved
- Recovering/Resolving
- Not Recovered/Not Resolved
- Recovered/Resolved with Sequelae
- Fatal
- Unknown

Any non-serious adverse event which occurs in the course of the study should be monitored and followed for up to 30 days following the last dose of test material.

7.4 Female Partner Pregnancy Reporting

The investigator will attempt to collect pregnancy information on any female partner of a male study subject who becomes pregnant while participating in this study. The investigator will record pregnancy information on the appropriate form and submit it to Sirtris and Akos within 2 weeks of learning of the partner's pregnancy. The partner will also be followed to determine the outcome of the pregnancy. Information on the status of the mother and child will be forwarded to Sirtris and Akos. Generally, follow-up will be no longer than 6 to 8 weeks following the estimated delivery date. Any premature termination of the pregnancy will be reported.

8 STATISTICAL PROCEDURES

There are no formal calculations of power or sample size for this study. The sample size has been chosen based on feasibility to allow preliminary characterisation of safety, tolerability and pharmacokinetics and to explore pharmacodynamic measures.

Alphas will be two-tailed in nature and set at 0.05 for all analyses unless otherwise stated. As this is an exploratory study, no adjustment for multiple analyses will be made.

Exploratory analyses will test for a relation between activity levels assessed from pedometer readings and changes in measures in subjects receiving SRT2104 relative to placebo.

8.1 Randomization and Stratification

On entry into the study subjects will be randomized to SRT2104 2.0 g/day, SRT2104 0.5g/day or placebo using a predetermined randomization schedule. All subjects will receive the assigned, double blinded study medication for 28 days.

8.2 Populations for Analysis

Safety: Subjects who are treated with any amount of test material will be included in the safety population. Safety data summaries and analyses will be produced for this population.

Pharmacokinetics: Subjects who receive a dose of SRT2104 and have pharmacokinetic data available will be included in the pharmacokinetics population, with data summarized by dose and gender.

Efficacy: Subjects who are randomized, receive study agent, and have at least one post baseline efficacy assessment will be included in efficacy analyses.

8.3 Procedures for Handling Missing, Unused, and Spurious Data

All available data will be included in data listings and tabulations. No imputation of values for missing data will be performed.

Percentages of subjects with AEs or laboratory toxicities will be based on non-missing values.

Data that are potentially spurious or erroneous will be examined under the auspices of standard data management operating procedures.

8.4 Statistical Methods

8.4.1 Safety Analysis

Safety evaluations will be based on the incidence, intensity and type of AEs and clinically significant changes in the subject's physical examination findings, vital signs, ECG studies, and clinical laboratory results. Safety variables will be tabulated and presented for all subjects who receive SRT2104 (i.e., the safety population).

Exposure to SRT2104 and reasons for discontinuation of study treatment will be tabulated.

AEs will be coded using the Medical Dictionary of Regulatory Activities (MedDRA) AE coding system for purposes of summarization. All AEs occurring on study will be listed in by-subject data listings. Treatment-emergent events will be tabulated, where treatment-emergent is defined as any AE that occurs after administration of the first dose of test material through 30 days after the last dose of SRT2104, any event that is considered causally drug-related regardless of the start date of the event, or any event that is present at baseline but worsens in intensity or is subsequently considered drug-related by the investigator. Events that are considered related to treatment (unlikely related, possibly related, or related) will also be tabulated. Tabulation will also be provided that enumerates AEs by maximum severity. Deaths, other SAEs, and events resulting in study discontinuation will be tabulated.

Change from baseline in clinical laboratory parameters will be summarized across time on study. Shift tables may be produced for selected laboratory parameters if implied by the data. Changes in vital sign parameters will be summarized over time in a similar fashion to laboratory parameters, and any abnormal values will be tabulated.

Additional safety analyses may be determined at any time without prejudice, in order to most clearly enumerate rates of toxicities and to further define the safety profile of SRT2104.

Blood urea nitrogen (BUN) will be calculated from the urea laboratory values and tabulated in the tables, listings and figures.

8.4.2 Pharmacokinetic (PK) Analysis

Subjects with partial data will be evaluated on a case-by-case basis to determine if sufficient data are available for meaningful analysis. Values excluded from the pharmacokinetic analysis will be flagged with an asterisk and concentration values reported as below the level of quantification

(BLQ) will be listed with the lower limit of quantification (LLQ) in parenthesis or as footnote. Pre-dose sample concentrations that are BLQ or missing will be assigned a numerical value of zero for further PK calculations. Plasma concentration values for sample data points with missing data occurring prior to the last quantifiable data point will not be interpolated other than those missing at the beginning (t=0 hour) or end (t=24 hour) of the dosing interval. If the concentration at time of dosing (t=0 hour) is missing, then the concentration is set to equal the concentration at the end of the dosing interval (assuming linear pharmacokinetics). Non-compartmental pharmacokinetic analysis will be performed using WinNonLin v5.2 (Pharsight).

Statistical Analysis of Pharmacokinetics

The statistical analysis will be performed using SAS® for Windows (Version 9.1.3 or higher) (SAS Institute, Cary, North Carolina). Graphics will be prepared with SAS® for Windows, version 9.1.3, or higher (SAS Institute, Cary, NC). Listing of individual subject plasma SRT2104 concentrations, actual blood sampling times, and graphs of individual and mean concentration vs. time will be prepared. Descriptive statistics (N, mean, SD, CV%, median, minimum, and maximum) will be used to summarize SRT2104 concentration data at each planned sampling time point for each dose level. BLQ values will be set to zero prior to calculation of descriptive statistics for the plasma concentration-time profile.

Following logarithmic transformation the primary pharmacokinetic endpoints will be subjected to an analysis of variance (ANOVA) that will include terms in the model for sequence, subject within sequence, period, treatment and gender. For comparison, point estimates and 90% confidence intervals for the difference between each pair of required contrasts will be constructed using the residual mean square error obtained from the ANOVA. The point and interval estimates will then be back transformed to give estimates of the ratio of the geometric least squares means and 90% confidence intervals for the ratios in the comparison.

8.4.3 Efficacy Analysis

T-tests or non-parametric equivalents will be employed to assess the differences between placebo and the doses of SRT2104 in activity levels and muscle characteristics assessed in the study. Actual numbers, change and percent change will be used in these determination.

8.5 Procedures for Reporting Deviations to Original Statistical Analysis Plan

A formal statistical analysis plan for the analysis and presentation of data from this study will be prepared before database lock. Deviations from the statistical analyses outlined in this protocol will be indicated in this plan; any further modifications will be noted in the final clinical study report.

9 ADMINISTRATIVE REQUIREMENTS

9.1 Good Clinical Practice

The study will be conducted in accordance with the ICH for GCP and the appropriate regulatory requirement(s). The investigator will be thoroughly familiar with the appropriate use of the study drug as described in the protocol and Investigator's Brochure. Essential clinical documents will be maintained to demonstrate the validity of the study and the integrity of the data collected. The Investigator Site File and associated study documentation will be archived for up to 15 years. The study documentation may be transferred to an offsite storage facility during this period but will remain under the control of the site. The sponsor will delegate the set up and maintenance of the Sponsor Trial Master File (TMF). The TMF will be returned to the sponsor at the end of the study, who will archive it for up to 15 years.

9.2 Ethical Considerations

The study will be conducted in accordance with ethical principles founded in the Declaration of Helsinki (see Appendix Section 12.1). The IRB/ IEC will review all appropriate study documentation in order to safeguard the rights, safety and well being of the subjects. The study will only be conducted at sites where IRB/IEC approval has been obtained. The protocol, Investigator's Brochure, informed consent, advertisements (if applicable), written information given to the subjects, safety updates, annual progress reports, and any revisions to these documents will be provided to the IRB/IEC by the investigator.

9.3 Subject Information and Informed Consent

After the study has been fully explained, written informed consent will be obtained from either the subject or their guardian or legal representative prior to study participation. The method of obtaining and documenting the informed consent and the contents of the consent will comply with ICH-GCP and all applicable regulatory requirement(s).

9.4 Subject Confidentiality

In order to maintain subject privacy, all CRFs, study drug accountability records, study reports and communications will identify the subject by initials and the assigned Subject number. The investigator will grant monitor(s) and auditor(s) from Sirtris or its designee and regulatory authority(ies) access to the subject's original medical records for verification of data gathered on

the CRFs and to audit the data collection process. The subject's confidentiality will be maintained and will not be made publicly available to the extent permitted by the applicable laws and regulations.

9.5 Protocol Compliance

The investigator will conduct the study in compliance with the protocol provided by Sirtris, and given approval/favorable opinion by the Research Ethics Committee (REC) and the MHRA. Modifications to the protocol should not be made without agreement by both the investigator and Sirtris. Changes to the protocol will require written approval/favorable opinion from the REC/MHRA prior to implementation, except when the modification is needed to eliminate an immediate hazard(s) to subjects. Sirtris will submit all protocol modifications to the MHRA in accordance with the governing regulations.

When immediate deviation from the protocol is required to eliminate an immediate hazard(s) to subjects, the investigator will contact Sirtris, if circumstances permit, to discuss the planned course of action. Any departures from the protocol must be fully documented in the CRF and source documentation.

9.6 Study Monitoring

Monitoring and auditing procedures developed by Sirtris will be followed, in order to comply with GCP guidelines. On-site checking of the CRFs for completeness and clarity, cross-checking with source documents, and clarification of administrative matters will be performed.

The study will be monitored by Sirtris or its designee. Monitoring will be done by personal visits from a representative of the sponsor (site monitor) who will review the CRFs and source documents. The site monitor will ensure that the investigation is conducted according to protocol design and regulatory requirements by frequent communications (letter, telephone, and fax).

All unused study drug and other study materials are to be returned to Sirtris or destroyed on site in the witness of a monitor after the clinical phase of the study has been completed (see Section 9.9).

9.7 On-site Audits

Regulatory authorities, the IEC/IRB, and/or Sirtris' clinical quality assurance group may request access to all source documents, CRFs, and other study documentation for on-site audit or

inspection. Direct access to these documents must be guaranteed by the investigator, who must provide support at all times for these activities.

9.8 Case Report Form Completion

Case report forms will be completed for each study subject. It is the investigator's responsibility to ensure the accuracy, completeness, legibility and timeliness of the data reported in the subject's CRF. Source documentation supporting the CRF data should indicate the subject's participation in the study and should document the dates and details of study procedures, adverse events and subject status.

The investigator, or designated representative, should complete the CRF pages as soon as possible after information is collected, preferably on the same day that a study subject is seen for an examination, treatment, or any other study procedure. Any outstanding entries must be completed immediately after the final examination. An explanation should be given for all missing data.

9.9 Drug Accountability

Accountability for the study drug at the study site and with the subject is the responsibility of the investigator. The investigator will ensure that the study drug is used only in accordance with this protocol. Where allowed, the investigator may choose to assign some of the drug accountability responsibilities to a pharmacist or other appropriate individual. Drug accountability records indicating the drug's delivery date to the site, inventory at the site, use by each subject, and return to Sirtris (or destruction and disposal of the drug, if approved by Sirtris) will be maintained by the clinical site. These records will adequately document that the subjects were provided the doses as specified in the protocol and should reconcile all study drug received from Sirtris. Accountability records will include dates, quantities, batch/serial numbers, expiration dates (if applicable), and Subject numbers. The sponsor or its designee will assign a monitor to review drug accountability at the site on an ongoing basis during monitoring visits.

All unused and used study drug will be retained at the site until they are inventoried by the monitor. All used, unused or expired study drug will be returned to Sirtris or if authorized, disposed of at the study site and documented. All material containing SRT2104 will be treated and disposed of as hazardous waste in accordance with governing regulations.

9.10 Premature Closure of the Study

This study may be prematurely terminated, if in the opinion of the investigator or Sirtris, there is sufficient reasonable cause. Written notification documenting the reason for study termination will be provided to the investigator or Sirtris by the terminating party.

Circumstances that may warrant termination include, but are not limited to:

- Determination of unexpected, significant, or unacceptable risk to subjects
- Failure to enter subjects at an acceptable rate
- Insufficient adherence to protocol requirements
- Insufficient complete and/or evaluable data
- Plans to modify, suspend or discontinue the development of the study drug

Should the study be closed prematurely, all study materials must be returned to Sirtris.

9.11 Record Retention and Publication Policy

The investigator will maintain all study records according to ICH-GCP, applicable regulatory requirement(s) and Sirtris' record retention policy. Records will be retained for at least five years after the last marketing application approval or five years after formal discontinuation of the clinical development of the investigational product or according to applicable regulatory requirement(s). The study documentation may be transferred to an offsite storage facility during this period but will remain under the control of the site. If the investigator withdraws from the responsibility of keeping the study records, custody must be transferred to a person willing to accept the responsibility. Sirtris must be notified in writing in advance of any change in disposition of the study records, including if a custodial change occurs.

Publication of the results of the study, whether in whole or in part, shall be within the sole and absolute discretion of Sirtris Pharmaceuticals, Inc. Any CROs, individuals, or organizations contracted by Sirtris shall not be entitled to publish any of the data or information arising during or out of the provision of the services without the prior written consent of Sirtris Pharmaceuticals. For the avoidance of doubt, Sirtris Pharmaceuticals reserves the unqualified right to reject any paper or

article utilizing any data generated from CROs, individuals, or organizations contracted by Sirtris before such paper or article is presented or submitted for publication.

9.12 Liability and Insurance

Sirtris will be subscribed to an insurance policy covering, in its terms and provisions, its legal liability for injuries caused to participating persons and arising out of this research performed strictly in accordance with the scientific protocol as well as with applicable law and professional standards.

10 USE OF INFORMATION

All information regarding SRT2104 supplied by Sirtris to the investigator is privileged and confidential information. The investigator agrees to use this information to accomplish the study and will not use it for other purposes without consent from Sirtris. It is understood that there is an obligation to provide Sirtris with complete data obtained during the study. The information obtained from the clinical study will be used towards the development of SRT2104 and may be disclosed to regulatory authority(ies), other investigators, corporate partners, or consultants as required.

Publication of the results of the study, whether in whole or in part, shall be within the sole and absolute discretion of Sirtris Pharmaceuticals. Investigators, sites, CROs and/or designees shall not be entitled to publish any of the data or information arising during or out of the provision of the services without the prior written consent of Sirtris Pharmaceuticals. For the avoidance of doubt Sirtris Pharmaceuticals reserves the unqualified right to reject any paper or article utilizing any data generated from this study before such paper or article is presented or submitted for publication.

11 INVESTIGATOR AGREEMENT

I have read the Protocol entitled, "**A randomized, placebo-controlled, double-blind, clinical study to assess the safety, tolerability and pharmacokinetics of oral SRT2104 capsules administered to healthy elderly subjects for 28 days"**

I agree to conduct the study as detailed herein and in compliance with ICH Guidelines for Good Clinical Practice and applicable regulatory requirements and to inform all who assist me in the conduct of this study of their responsibilities and obligations.

____
Principal investigator printed name

____ __________________
Principal investigator signature Date

____
____
____
Investigational site or name of institution and location (printed)

12 REFERENCES

12.1 Declaration of Helsinki

World Medical Association Declaration of Helsinki: Ethical Principles for Medical Research Involving Human Subjects.

Adopted by the 18th WMA General Assembly, Helsinki, Finland, June 1964 and amended by the 29th WMA General Assembly, Tokyo, Japan, October 1975, 35th WMA General Assembly, Venice, Italy, October 1983, 41st WMA General Assembly, Hong Kong, September 1989, 48th WMA General Assembly, Somerset West, Republic of South Africa, October 1996 and the 52nd WMA General Assembly, Edinburgh, Scotland, October 2000. Note of clarification on Paragraph 29 added by the WMA General Assembly, Washington 2002. Note of Clarification on Paragraph 30 added by the WMA General Assembly, Tokyo 2004. Amended by the WMA General Assembly, Seoul, October 2008.

A INTRODUCTION

1. The World Medical Association (WMA) has developed the Declaration of Helsinki as a statement of ethical principles for medical research involving human subjects, including research on identifiable human material and data.

   The Declaration is intended to be read as a whole and each of its constituent paragraphs should not be applied without consideration of all other relevant paragraphs.
2. Although the Declaration is addressed primarily to physicians, the WMA encourages other participants in medical research involving human subjects to adopt these principles.
3. It is the duty of the physician to promote and safeguard the health of patients, including those who are involved in medical research. The physician's knowledge and conscience are dedicated to the fulfilment of this duty.
4. The Declaration of Geneva of the WMA binds the physician with the words, "The health of my patient will be my first consideration," and the International Code of Medical Ethics declares that, "A physician shall act in the patient's best interest when providing medical care."
5. Medical progress is based on research that ultimately must include studies involving human subjects. Populations that are underrepresented in medical research should be provided appropriate access to participation in research.
6. In medical research involving human subjects, the well-being of the individual research subject must take precedence over all other interests.
7. The primary purpose of medical research involving human subjects is to understand the causes, development and effects of diseases and improve preventive, diagnostic and therapeutic interventions (methods, procedures and treatments). Even the best current interventions must be evaluated continually through research for their safety, effectiveness, efficiency, accessibility and quality.
8. In medical practice and in medical research, most interventions involve risks and burdens.

9. Medical research is subject to ethical standards that promote respect for all human subjects and protect their health and rights. Some research populations are particularly vulnerable and need special protection. These include those who cannot give or refuse consent for themselves and those who may be vulnerable to coercion or undue influence.
10. Physicians should consider the ethical, legal and regulatory norms and standards for research involving human subjects in their own countries as well as applicable international norms and standards. No national or international ethical, legal or regulatory requirement should reduce or eliminate any of the protections for research subjects set forth in this Declaration.

B BASIC PRINCIPLES FOR ALL MEDICAL RESEARCH

11. It is the duty of physicians who participate in medical research to protect the life, health, dignity, integrity, right to self-determination, privacy, and confidentiality of personal information of research subjects.
12. Medical research involving human subjects must conform to generally accepted scientific principles, be based on a thorough knowledge of the scientific literature, other relevant sources of information, and adequate laboratory and, as appropriate, animal experimentation. The welfare of animals used for research must be respected.
13. Appropriate caution must be exercised in the conduct of medical research that may harm the environment.
14. The design and performance of each research study involving human subjects must be clearly described in a research protocol. The protocol should contain a statement of the ethical considerations involved and should indicate how the principles in this Declaration have been addressed. The protocol should include information regarding funding, sponsors, institutional affiliations, other potential conflicts of interest, incentives for subjects and provisions for treating and/or compensating subjects who are harmed as a consequence of participation in the research study. The protocol should describe arrangements for post-study access by study subjects to interventions identified as beneficial in the study or access to other appropriate care or benefits.
15. The research protocol must be submitted for consideration, comment, guidance and approval to a research ethics committee before the study begins. This committee must be independent of the researcher, the sponsor and any other undue influence. It must take into consideration the laws and regulations of the country or countries in which the research is to be performed as well as applicable international norms and standards but these must not be allowed to reduce or eliminate any of the protections for research subjects set forth in this Declaration. The committee must have the right to monitor ongoing studies. The researcher must provide monitoring information to the committee, especially information about any serious adverse events. No change to the protocol may be made without consideration and approval by the committee.
16. Medical research involving human subjects must be conducted only by individuals with the appropriate scientific training and qualifications. Research on patients or healthy volunteers requires the supervision of a competent and appropriately qualified physician or other health care professional. The responsibility for the protection of research subjects must always rest

with the physician or other health care professional and never the research subjects, even though they have given consent.

17. Medical research involving a disadvantaged or vulnerable population or community is only justified if the research is responsive to the health needs and priorities of this population or community and if there is a reasonable likelihood that this population or community stands to benefit from the results of the research.
18. Every medical research study involving human subjects must be preceded by careful assessment of predictable risks and burdens to the individuals and communities involved in the research in comparison with foreseeable benefits to them and to other individuals or communities affected by the condition under investigation.
19. Every clinical trial must be registered in a publicly accessible database before recruitment of the first subject.
20. Physicians may not participate in a research study involving human subjects unless they are confident that the risks involved have been adequately assessed and can be satisfactorily managed. Physicians must immediately stop a study when the risks are found to outweigh the potential benefits or when there is conclusive proof of positive and beneficial results.
21. Medical research involving human subjects may only be conducted if the importance of the objective outweighs the inherent risks and burdens to the research subjects.
22. Participation by competent individuals as subjects in medical research must be voluntary. Although it may be appropriate to consult family members or community leaders, no competent individual may be enrolled in a research study unless he or she freely agrees.
23. Every precaution must be taken to protect the privacy of research subjects and the confidentiality of their personal information and to minimize the impact of the study on their physical, mental and social integrity.
24. In medical research involving competent human subjects, each potential subject must be adequately informed of the aims, methods, sources of funding, any possible conflicts of interest, institutional affiliations of the researcher, the anticipated benefits and potential risks of the study and the discomfort it may entail, and any other relevant aspects of the study. The potential subject must be informed of the right to refuse to participate in the study or to withdraw consent to participate at any time without reprisal. Special attention should be given to the specific information needs of individual potential subjects as well as to the methods used to deliver the information. After ensuring that the potential subject has understood the information, the physician or another appropriately qualified individual must then seek the potential subject's freely-given informed consent, preferably in writing. If the consent cannot be expressed in writing, the non-written consent must be formally documented and witnessed.
25. For medical research using identifiable human material or data, physicians must normally seek consent for the collection, analysis, storage and/or reuse. There may be situations where consent would be impossible or impractical to obtain for such research or would pose a threat to the validity of the research. In such situations the research may be done only after consideration and approval of a research ethics committee.
26. When seeking informed consent for participation in a research study the physician should be particularly cautious if the potential subject is in a dependent relationship with the physician or

may consent under duress. In such situations the informed consent should be sought by an appropriately qualified individual who is completely independent of this relationship.

27. For a potential research subject who is incompetent, the physician must seek informed consent from the legally authorized representative. These individuals must not be included in a research study that has no likelihood of benefit for them unless it is intended to promote the health of the population represented by the potential subject, the research cannot instead be performed with competent persons, and the research entails only minimal risk and minimal burden.
28. When a potential research subject who is deemed incompetent is able to give assent to decisions about participation in research, the physician must seek that assent in addition to the consent of the legally authorized representative. The potential subject's dissent should be respected.
29. Research involving subjects who are physically or mentally incapable of giving consent, for example, unconscious patients, may be done only if the physical or mental condition that prevents giving informed consent is a necessary characteristic of the research population. In such circumstances the physician should seek informed consent from the legally authorized representative. If no such representative is available and if the research cannot be delayed, the study may proceed without informed consent provided that the specific reasons for involving subjects with a condition that renders them unable to give informed consent have been stated in the research protocol and the study has been approved by a research ethics committee. Consent to remain in the research should be obtained as soon as possible from the subject or a legally authorized representative.
30. Authors, editors and publishers all have ethical obligations with regard to the publication of the results of research. Authors have a duty to make publicly available the results of their research on human subjects and are accountable for the completeness and accuracy of their reports. They should adhere to accepted guidelines for ethical reporting. Negative and inconclusive as well as positive results should be published or otherwise made publicly available. Sources of funding, institutional affiliations and conflicts of interest should be declared in the publication. Reports of research not in accordance with the principles of this Declaration should not be accepted for publication.

C ADDITIONAL PRINCIPLES FOR MEDICAL RESEARCH COMBINED WITH MEDICAL CARE

31. The physician may combine medical research with medical care only to the extent that the research is justified by its potential preventive, diagnostic or therapeutic value and if the physician has good reason to believe that participation in the research study will not adversely affect the health of the patients who serve as research subjects.
32. The benefits, risks, burdens and effectiveness of a new intervention must be tested against those of the best current proven intervention, except in the following circumstances:
    - The use of placebo, or no treatment, is acceptable in studies where no current proven intervention exists; or

- Where for compelling and scientifically sound methodological reasons the use of placebo is necessary to determine the efficacy or safety of an intervention and the patients who receive placebo or no treatment will not be subject to any risk of serious or irreversible harm. Extreme care must be taken to avoid abuse of this option.

33. At the conclusion of the study, patients entered into the study are entitled to be informed about the outcome of the study and to share any benefits that result from it, for example, access to interventions identified as beneficial in the study or to other appropriate care or benefits.
34. The physician must fully inform the patient which aspects of the care are related to the research. The refusal of a patient to participate in a study or the patient's decision to withdraw from the study must never interfere with the patient-physician relationship.
35. In the treatment of a patient, where proven interventions do not exist or have been ineffective, the physician, after seeking expert advice, with informed consent from the patient or a legally authorized representative, may use an unproven intervention if in the physician's judgement it offers hope of saving life, re-establishing health or alleviating suffering. Where possible, this intervention should be made the object of research, designed to evaluate its safety and efficacy. In all cases, new information should be recorded and, where appropriate, made publicly available.

12.2 Literature References

ACSM. (2005) ACSM's Guidelines For Exercise Testing And Prescription ,
American College of Sports Medicine, Lippincott Williams and Wilkins

Baker G, Mutrie N, Lowry R. Using pedometers as motivational tools: are goals set in steps more effective than goals set in minutes for increasing walking? *International Journal of Health Promotion and Education.* 2008

Borg, G. (1998) "Borg's perceived exertion and pain scales." Champaign, IL.

Houmard JA, Weidner ML, Gavigan KE, Tyndall GL, Hickey MS, Alshami A. Fiber type and citrate synthase activity in the human gastrocnemius and vastus lateralis with aging. *J. Appl. Physiol.* 1998; 85:1337-41

Lagouge M, Argmann C, Gerhart-Hines, Meziane H, Lerin C, Daussin F, Messadeq N, Milne J, Lambert P, Elliott P, Geny B, Laakso M, Puigserver P, Auwerx J. Resveratrol improves mitochondrial function and protects against metabolic disease by activating SIRT-1 and PGC-1alpha. *Cell.* 2006; 127(6):1109-1122

Lanza IR, Short DK, Short KR, Raghavakainal S, Basu R, Joyner MJ, McConnell JP, Nair KS. Endurance exercise as a countermeasure for aging. *Diabetes.* 2008; 57:2933-2942

Larson-Meyer DE, Newcomer BR, Hunter GR, Hetherington HP, Weinsier RL. 31P MRS measurement of mitochondrial function in skeletal muscle: reliability, force-level sensitivity and relation to whole body maximal oxygen uptake. NMR Biomed. 2000 Jan;13(1):14-27.

Larson-Meyer DE, Newcomer BR, Hunter GR, Joanisse DR, Weinsier RL, Bamman MM. Relation between *in vivo* and *in vitro* measurements of skeletal muscle oxidative metabolism. Muscle Nerve. 2001 Dec;24(12):1665-76.

Matthews PM, Allaire C, Shoubridge EA, Karpati G, Carpenter S, Arnold DL. *In vivo* muscle magnetic resonance spectroscopy in the clinical investigation of mitochondrial disease. Neurology. 1991 Jan;41(1):114-20.

Milne JC, Lambert PD, Schenk S, Carney DP, Smith JJ, Gagne DJ, Jin L, Boss O, Perni RB, Vu CB. Small molecule activators of SIRT1 as therapeutics for the treatment of type 2 diabetes. *Nature.* 2007; 450:712-716

Murase T, Haramizu S, Ota N, Hase T. Suppression of the aging-associated decline in physical performance by a combination of resveratrol intake and habitual exercise in senescence-accelerated mice. Biogerontology. 2008 Oct 1. Published electronically ahead of print.

Schocke MF, Esterhammer R, Kammerlander C, Rass A, Kremser C, Fraedrich G,Jaschke WR, Greiner A. High-energy phosphate metabolism during incremental calf exercise in humans measured by 31 phosphorus magnetic resonance spectroscopy (31PMRS). Magn Reson Imaging. 2004 Jan;22(1):109-15.

Schroeder ET, Singh A, Bhasin S, Storer TW, Azen C, Davidson T, Martinez C, Sinhahikim I, Jaque SV, Terk M, Sattler FR. Effects of an oral androgen on muscle and metabolism in older, community-dwelling men. *Am. J. Physiol. Endocrinol. Metab.* 2002; 284:120-128.

Schroeder ET, Terk M, Sattler FR. Androgen therapy improves muscle mass and strength but not muscle quality: results from two studies. *Am. J. Physiol. Endocrinol. Metab.* 2003; 285:16-24.

Sorock GS, Bush TL, Golden AL, Fried LP, Breuer B, Hale WE. Physical activity and fracture risk in a free-living elderly cohort. *J. Gerontol.* 1988; 43(5):134-139.

Tully MA, Cupples ME, Hart ND, McEneny J, McGlade KJ, Chan WS, Young IS. Randomised controlled trial of home-based walking programmes at and below current recommended levels of exercise in sedentary adults. J Epidemiol Community Health. 2007 Sep;61(9):778-83.

11 INVESTIGATOR AGREEMENT

I have read the Protocol entitled, "**A randomized, placebo-controlled, double-blind, clinical study to assess the safety, tolerability and pharmacokinetics of oral SRT2104 capsules administered to healthy elderly subjects for 28 days**"

I agree to conduct the study as detailed herein and in compliance with ICH Guidelines for Good Clinical Practice and applicable regulatory requirements and to inform all who assist me in the conduct of this study of their responsibilities and obligations.

N. VINCENZO LIBRI

Principal investigator printed name

[signature] 09-12-2009

Principal investigator signature Date

SIR JOHN McMICHAEL CLINICAL RESEARCH CENTRE

IMPERIAL COLLEGE NHS TRUST

HAMMERSMITH HOSPITAL - DU CANE ROAD, LONDON W12 0HS - UK -

Investigational site or name of institution and location (printed)
