# Supplementary figures and images for: A Pilot Randomized, Placebo Controlled, Double Blind Phase I Trial of the Novel SIRT1 Activator SRT2104 in Elderly Volunteers
Source: PLoS One. 2012 Dec 20;7(12):e51395. doi: 10.1371/journal.pone.0051395 (PMC3527451; doi:10.1371/journal.pone.0051395)

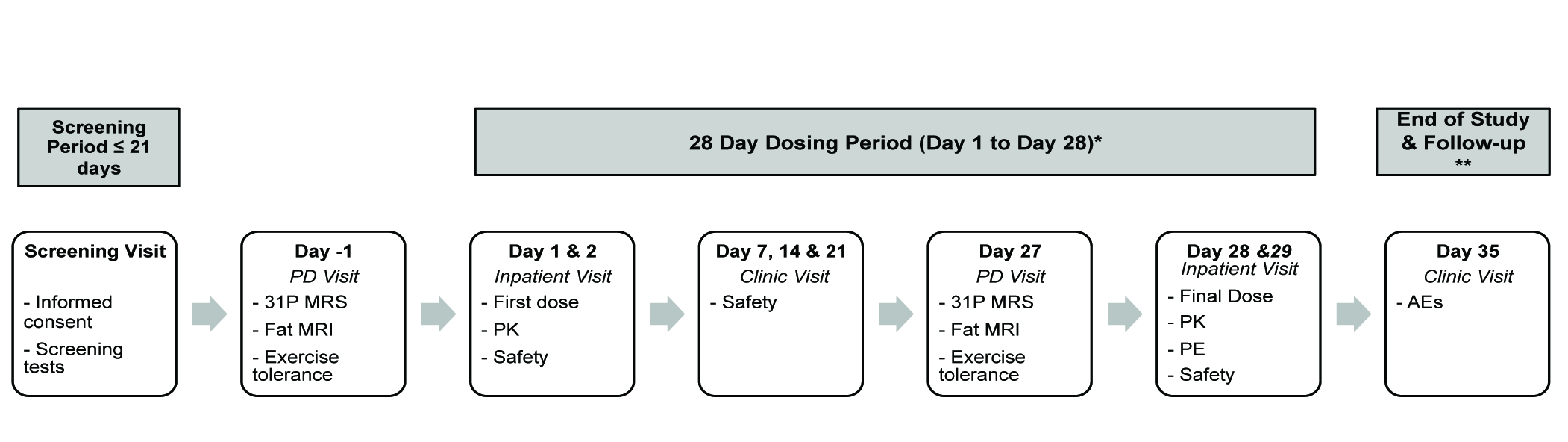

Supplement: Figure S1 — Study design schematic. Subjects underwent a screening period within 21 days of the first dose. Subjects received a single daily dose of SRT2104 for 28 days and had pharmacodynamic study visits at day −1 and day 27. [* Telephone safety assessments were made approximately on days 3, 5, 10, 17, 20, 24; ** An end of study telephone safety assessment was made approximately on day 58]. (TIF) [file pone.0051395.s004.tif]

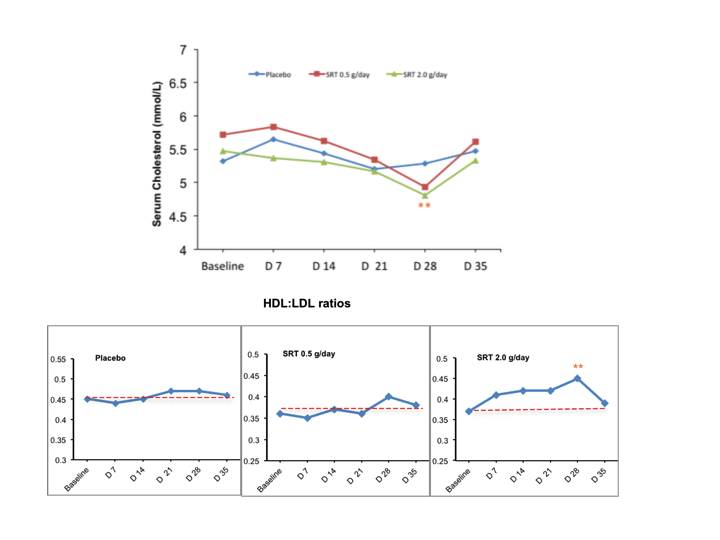

Supplement: Figure S2 — Total serum cholesterol and HDL: LDL ratios after placebo and SRT2104 treatments. ** Denote statistical significance. Changes from baseline in total cholesterol where statistically significant at both SRT2104 doses levels on day 28 relative to baseline (0.5 g/day, p = 0.007 and 2.0 g/day, p = 0.018). Changes in HDL:LDL ratios were statistically significant (p = 0.014) in the SRT2104 2.0 g/day group on day 28 relative to baseline. (TIFF) [file pone.0051395.s005.tiff]

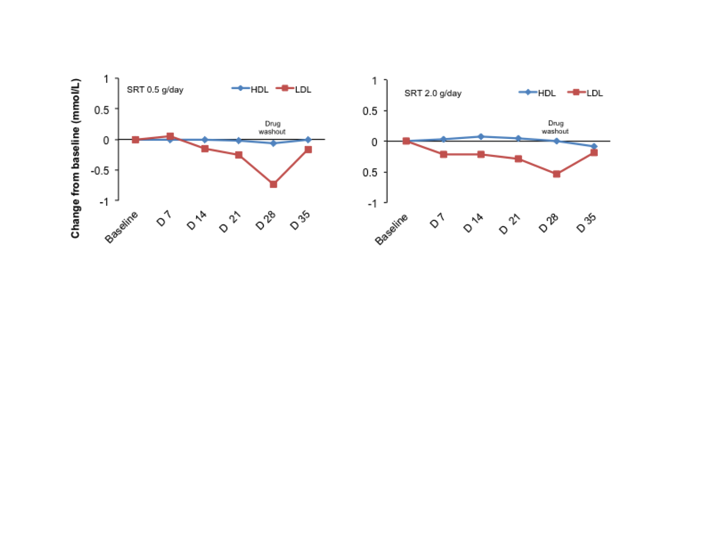

Supplement: Figure S3 — Time course of serum HDL and LDL cholesterol before and after 28-day treatment with SRT2104 at 0.5 g/day and 2.0 g/day doses. (TIFF) [file pone.0051395.s006.tiff]

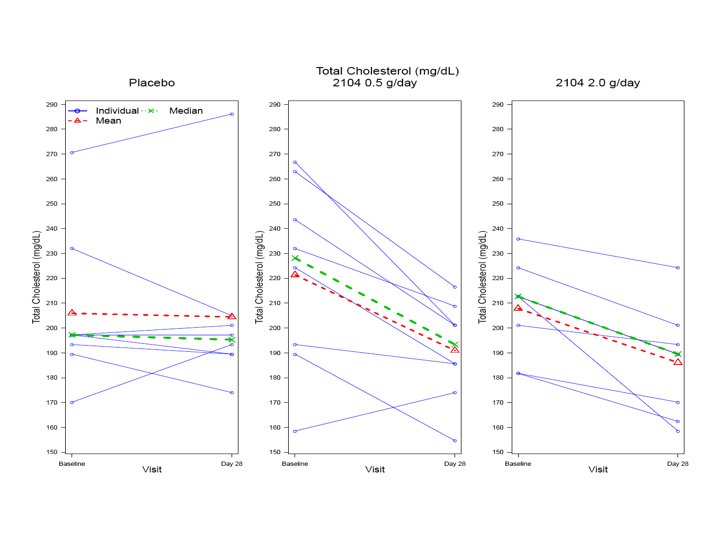

Supplement: Figure S4 — Individual serum cholesterol levels at baseline and after 28-day treatment with SRT2104 at 0.5 g/day and 2.0 g/day doses. (TIFF) [file pone.0051395.s007.tiff]
