# Supplementary material for: A Pilot Randomized, Placebo Controlled, Double Blind Phase I Trial of the Novel SIRT1 Activator SRT2104 in Elderly Volunteers
Source: PLoS One. 2012 Dec 20;7(12):e51395. doi: 10.1371/journal.pone.0051395 (PMC3527451; doi:10.1371/journal.pone.0051395)
Supplement: Table S1 — 31P ADP and PCr recovery time constant after exercise at day 27 mutations with HCC risk in the meta-analysis. (DOC) [file pone.0051395.s008.doc]

**Table S1. 31P ADP and PCr recovery time constant after exercise at day 27**

| **Dose group** | **Baseline mean (sec)** | | **Change from baseline mean (sec)** | | **Change from Baseline (p-values)** | |
| --- | --- | --- | --- | --- | --- | --- |
|  | **ADP T_1/2_** | **PCr T_1/2_** | **ADP T_1/2_** | **PCr T_1/2_** | **ADP T_1/2_** | **PCr T_1/2_** |
| Placebo  (SD) | 32  (4) | 40  (6) | 2  (8) | 2  (10) |  |  |
| 0.5g/day  (SD) | 29  (11) | 39  (17) | 0  (10) | -1  (18) | 0.089 | 0.373 |
| 2.0g/day  (SD) | 33  (7) | 39  (10) | -2  (6) | -4  (7) | 0.197 | 0.083 |
