# Supplementary material for: A Pilot Randomized, Placebo Controlled, Double Blind Phase I Trial of the Novel SIRT1 Activator SRT2104 in Elderly Volunteers
Source: PLoS One. 2012 Dec 20;7(12):e51395. doi: 10.1371/journal.pone.0051395 (PMC3527451; doi:10.1371/journal.pone.0051395)
Supplement: Table S2 — Time to cessation (TTC) and rated perceived exertion (RPE) in the incremental cycle ergometer test. (DOC) [file pone.0051395.s009.doc]

**Table S2. Time to cessation (TTC) and rated perceived exertion (RPE) in the incremental cycle ergometer test**

| **Dose group** | **Baseline mean** | | **Mean change from**  **baseline** | | **Change from baseline**  **(p-values)** | |
| --- | --- | --- | --- | --- | --- | --- |
|  | **TTC (sec)** | **RPE** | **TTC**  **(sec)** | **RPE** | **TTC**  **(sec)** | **RPE** |
| Placebo  Mean  (SD)  Range | 710 (177)  540-945 | 7  (2)  4-10 | 36  (34)  -1.0-96 | 0  (2)  -3-3 |  |  |
| 0.5g/day  Mean  (SD)  Range | 715  (180)  453-960 | 7  (3) | -31  (50)  -106-67 | 0  (3)  -3-6 | 0.004 | 0.962 |
| 2.0g/day  Mean  (SD)  Range | 691  (120)  480-850 | 8  (2) | 9  (27)  -29- 45 | -1  (2)  -3-1 | 0.292 | 0.638 |
